# Decoding depression: a comprehensive multi-cohort exploration of blood DNA methylation using machine learning and deep learning approaches

**DOI:** 10.1038/s41398-024-02992-y

**Published:** 2024-07-15

**Authors:** Aleksandr V. Sokolov, Helgi B. Schiöth

**Affiliations:** Department of Surgical Sciences, Functional Pharmacology and Neuroscience, Uppsala University, Uppsala, Sweden

**Keywords:** Diagnostic markers, Depression

## Abstract

The causes of depression are complex, and the current diagnosis methods rely solely on psychiatric evaluations with no incorporation of laboratory biomarkers in clinical practices. We investigated the stability of blood DNA methylation depression signatures in six different populations using six public and two domestic cohorts (n = 1942) conducting mega-analysis and meta-analysis of the individual studies. We evaluated 12 machine learning and deep learning strategies for depression classification both in cross-validation (CV) and in hold-out tests using merged data from 8 separate batches, constructing models with both biased and unbiased feature selection. We found 1987 CpG sites related to depression in both mega- and meta-analysis at the nominal level, and the associated genes were nominally related to axon guidance and immune pathways based on enrichment analysis and eQTM data. Random forest classifiers achieved the highest performance (AUC 0.73 and 0.76) in CV and hold-out tests respectively on the batch-level processed data. In contrast, the methylation showed low predictive power (all AUCs < 0.57) for all classifiers in CV and no predictive power in hold-out tests when used with harmonized data. All models achieved significantly better performance (>14% gain in AUCs) with pre-selected features (selection bias), with some of the models (joint autoencoder-classifier) reaching AUCs of up to 0.91 in the final testing regardless of data preparation. Different algorithmic feature selection approaches may outperform *limma*, however, random forest models perform well regardless of the strategy. The results provide an overview over potential future biomarkers for depression and highlight many important methodological aspects for DNA methylation-based depression profiling including the use of machine learning strategies.

## Introduction

Depression is a complex psychiatric condition influenced by many factors, such as life experiences [1], interpersonal relationships [2], and biological determinants, such as genetics [3–7], epigenetics [8–12], and expression profiles [13, 14]. This prevalent disorder, affecting up to 20% of the population [15, 16] poses a significant burden on healthcare systems globally. Presently, clinical structured interviews, based on criteria outlined in the Diagnostic and Statistical Manual of Mental Disorders 5th edition (DSM-5) [16], along with tools like the Beck Depression Inventory (BDI) [17], are employed to diagnose depression and its classical form, Major Depressive Disorder (MDD). Unfortunately, there are currently no clinically applicable lab-based techniques for the identification or validation of depression. This absence of reliable methods introduces major challenges in distinguishing patients with MDD from those experiencing temporary low mood or individuals with other psychiatric disorders exhibiting similar symptoms, such as bipolar disorder or anxiety disorder.

**Fig. 1:**
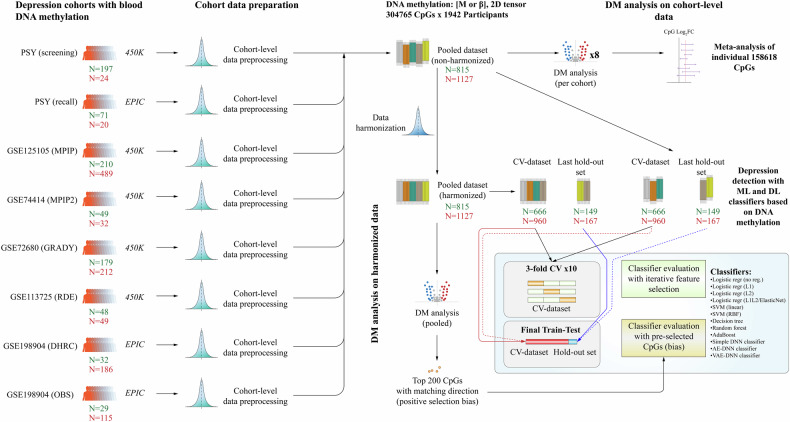
The workflow. This figure shows the workflow of the present work. Data from eight datasets (cohorts) was preprocessed and filtered at the cohort level. Red number indicates depressed cases, whereas green number indicates controls. The harmonized data was used to perform pooled differential methylation analysis, whereas the non-harmonized data was used to perform differential methylation analysis in the individual cohorts followed by meta-analysis of estimated effects. Both harmonized and non-harmonized data were used for classifier evaluation. Classifier evaluation was performed with 10 repetitions of threefold cross-validation and with a testing hold-out set representing independent batches. All evaluations were performed either with unbiased feature selection or with a pre-selected list of CpGs. CV cross-validation, ML machine learning, DL deep learning, DM differential methylation, AE auto-encoder, VAE variational autoencoder, DNN deep neural network.

Many genetic, epigenetic, and transcriptome studies have produced significant quantities of data on depression. For instance, genome-wide association studies (GWASs) have identified multiple single nucleotide polymorphisms (SNPs) associated with depression and are typically included in the GWAS catalog [4, 18]. Similarly, DNA methylation (DNAm) [19–24] and transcriptome [14] studies on depression have identified numerous potential markers. Meanwhile, the rapid development of suitable hardware and advances in research in the area of machine learning (ML) and deep learning (DL) have resulted in growing applications of such models in various fields, including life sciences and medicine [25–29]. As of today, only a few studies investigated the application of ML algorithms for depression detection using blood biomarkers, showing varying performances [30, 31]. Several studies explored the possibility of using DL frameworks with DNA methylation data to predict/characterize various conditions, such as cancer or Alzheimer’s disease, and others, showing promising predictive power [32–35]. In depression, however, the predictive power of blood DNA methylation was tested in isolated cohorts and with a limited number of ML classifiers yielding moderate performances (AUCs from 0.54 to 0.72) [36–38], with the largest evaluation being performed in a large single cohort from Scotland, using Lasso-regression-derived depression methylation score [37]. To our knowledge, there are no studies investigating the stability of DNA methylation depression features across different cohorts, populations, depression characterization methods as well as comparing performances of multiple classification algorithms. Thus, in this work, we identified depression-related methylation markers in eight cohorts (6 different populations) and explored the possibility of using blood DNA methylation for depression identification with ML frameworks [39], multilayer perceptrons (MLPs) [40], and autoencoders [41]. The outline of the present work is shown in Fig. 1.

## Materials and methods

Due to the extensive amount of the methods used, the main text contains main aspects of the methods, whereas the extensive description is provided in the supplementary materials (Data S1). The source code for all stages of the work is publicly available at https://github.com/AleksandrVSokolov/depression_ ML_DL.

### Ethics declaration

The present work uses data from eight studies on human subjects. The Psychiatric Health in Adolescent Study (PSY cohort) was conducted in Uppsala, Sweden, and was approved by the Regional Ethics Committee of Uppsala. All participants gave their written informed consent for participation. Records from other studies were deposited in the publicly available repository Gene Expression Omnibus (GEO) [42] and were approved by corresponding national ethical committees. Further information is available in the corresponding GEO records and associated publications: GSE125105 [43–46], GSE72680 [43, 47], GSE113725 [48], GSE198904 [23, 49, 50], GSE74414 [51].

### Cohorts

DNA methylation (DNAm) data for the present study was obtained from eight cohorts: PSY (screening), PSY (recall), Max Planck Institute of Psychiatry cohort 1 (GSE125105_MPIP) [43–46], Max Planck Institute of Psychiatry cohort 2 (GSE74414_MPIP2) [51], Grady Trauma Project cohort (GSE72680_GRADY) [43, 47], Royal Devon and Exeter (RDE) cohort (GSE113725_RDE) [48], Molecular Biomarkers of Antidepressant Response cohort (GSE198904_DHRC) [49, 50], and Observational clinical study cohort NCT02489305 (GSE198904_OBS) [23]. Listed cohorts were identified using advanced search on GEO with three different search queries: (((depression) AND methylation) AND Homo sapiens[Organism]); (((MDD) AND methylation) AND Homo sapiens[Organism]); (((depressed) AND methylation) AND Homo sapiens[Organism]). Only studies with more than 50 samples were included. The data for the PSY cohort has been already available for the research group. The demographic characteristics of all depression cohorts is available in Data S2. Description of the cohort data preparation and depression characterization per cohort could be found in Data S1.

### DNA methylation data preprocessing

DNAm data obtained as raw IDAT files for PSY and GSE125105_MPIP, and in CSV format for other cohorts were loaded and preprocessed using the *minfi* R package [52]. Subsequent processing involved background correction (some of the cohorts), quantile normalization, and correction for type I and type II probe bias, using the Beta Mixture Quantile Dilation method [53]. Additional filtering steps included the removal of sex chromosome probes, SNP-related probes, and cross-reactive probes [54, 55]. Participant and CpG site filtering was based on detection *p*-values, and the bead batch effect correction was performed. A Houseman algorithm was used to estimate white peripheral blood cell heterogeneity [56–58]. We used a regression-based approach to adjust methylation values for cell-based heterogeneity [59]. Briefly, methylation value is regressed against estimated cell proportions using a linear model. The obtained residuals (that represent the variance unexplained by cell proportions) are then added to the mean value for a CpG to obtain cell-type adjusted methylation intensity. The analysis was limited to overlapping CpG sites between HumanMethylation450 and HumanMethylationEPIC that passed QC steps in all cohort batches. Thus, each participant is characterized with a 1D methylation tensor comprising 304,765 methylation values.

DNAm intensity could be represented as either beta-values ($\beta \in \left[\mathrm{0,1}\right]$) or as M-values ($M\in R$), where R denotes real numbers. The usage of beta- and M-values was considered as hyperparameters for models. Each of eight cohort batches of the data passed through data preprocessing and QC pipeline separately. Then, methylation values were ordered based on genomic positions (cumulative) and batches were concatenated together across CpGs that passed data preparation and QC in all batches. The resulting merged data represents a batch-level processed dataset. Ordering of CpGs based on genomic positions was performed to ensure that features are in the same order for every participant. Quantile normalization, and stabilization with ComBat using a cohort of origin variable as the batch was used to obtain the harmonized dataset. Data harmonization was evaluated through PCA on hypervariable CpGs with a beta value difference >0.2. The first two dimensions were plotted to visualize the distribution of samples with respect to a cohort of origin or depression status. Detailed description of DNA methylation data preparation could be found in Data S1.

### Differential methylation analyses

Differential methylation analyses were conducted in the R environment, employing both pooled analysis of merged data from all cohorts and meta-analysis of individual cohorts. Pooled analysis utilized the harmonized dataset, and differential methylation was assessed using the limma R package with linear models and T statistics moderated by an empirical Bayes framework [60]. Covariates such as age, sex, and study factor were included in the models with depression status as the main predictor and methylation at the corresponding CpG as the dependent variable. A directional agreement index was calculated for nominally significant CpGs, indicating the fraction of cohorts where the difference in median methylation between cases and controls had the same sign.

For the meta-analysis of individual cohorts, we utilized pre-harmonized data (batch-level preprocessing), conducting limma-based modeling on individual cohorts without the study factor (8 levels). Model covariates in *limma* included age and sex as these were only variables available in all existing datasets, whereas depression status (binary) was used as the main predictor. Nominally significant CpGs in at least one cohort were assessed for occurrences at a false discovery rate of 5%, and a directional agreement index was calculated. We performed a meta-analysis of log2 fold changes (Log2FC) for CpGs that were at least nominally significant in a single cohort batch. We considered this as a minimal requirement and indication that such a CpG would have a non-zero effect in the meta-analysis. The meta-analysis was performed for log2 fold changes of probes as this is the effect size of a CpG in *limma* output. First, standard errors (SE) for log2 fold changes were estimated directly from the limma fit object. Then, we used an R package *metafor* and function *rma.uni* to perform the modeling, utilizing a weighted random effects model. The *rma.uni* used log2 fold changes (vector of 8 values) and corresponding sampling variances (SE^2^, vector of 8 values) as input per each CpG. The amount of heterogeneity (${\tau }^{2}$) was estimated using the Sidik–Jonkman estimator, and study weights were determined through the inverse-variance method (*metafor* default*)* [61, 62]. Modeling was performed individually as one CpG at a time. *P*-values estimated in the meta-analysis were adjusted using the false discovery rate (FDR) method.

### Sensitivity analysis and functional analyses

As the initial phenotypic data does not have reported smoking status, we performed estimation of the smoking score from methylation intensities (before harmonization) to see which proportion of participants could be potential smokers in the analyzed dataset. We used the R package *EpiSmokEr* [63] to perform estimation of the smoking score based on the methodology initially proposed by Elliott et al. [64]. We used two score thresholds, both 17.55 (for European population) and 11.79 (for Asian population) as reported in the initial paper [64].

The list of overlapping nominally significant CpGs obtained from pooled and meta-analysis was analyzed for gene ontology enrichment using the missMethyl R package, accounting for gene-length bias and multi-mapped CpG [65]. Additionally, CpGs were assessed as expression quantitative trait methylation (eQTMs) at FDR < 5%, utilizing data from the BIOS QTL browser, with gene annotation modified to replace associations with genes from BIOS QTL.

The obtained list of overlapping nominally significant CpGs was also mapped to chromatin regulatory elements identified by Roadmap Epigenomics Consortium [66]. Mapping was performed based on two cell/tissue types and included E062 primary mononuclear cells (blood) and E073 prefrontal cortex. A blood sample was used to represent the same sample source as in the current study. Prefrontal cortex was used as it has been consistently linked to depression biology [67]. After every CpG was mapped to a corresponding regulatory element (per tissue), we performed an enrichment analysis of mappings obtained from 1987 CpGs in comparison to a mapping from all CpGs included in the study (304,765). The enrichment was performed, using R package *clusterProfiler*, and was done separately for up-regulated and down-regulated probes.

### Feature selection for depression classification

Due to the substantial initial feature-to-participant ratio, a feature selection process was implemented before training classification models. Initially, a list of 200 CpGs, representing the Top 200 CpGs from pooled differential methylation analysis with consistent direction across all cohorts, was generated for assessing the “*maximal theoretical performance*” of classifiers (positive bias). To perform unbiased evaluation, a feature selection procedure was integrated into the model training process, utilizing *limma* to identify the top 200 differentially methylated CpGs exclusively from training sets generated within each cross-validation iteration or from the entire validation dataset in the final testing. For regularized logistic regression models, performance evaluation included the Top 10,000 CpGs from *limma* to select the most relevant features.

We compared *limma* with alternative algorithm-based feature selection strategies implemented within the training of models (unbiased feature selection). These strategies were based on built-in functions of the *scikit-learn* [39] module and included variance threshold methods (Top 5%, 1%, or 0.1% of CpGs are used as features), selection of features based on models with L1 regularization (linear support vector classifier and logistic regression), selection based on ANOVA F-value, and selection of CpGs based on ExtraTrees classifier. Variance threshold-based selectors identify 15239, 3048, and 305 CpGs (features), respectively. L1-based, ANOVA-based, and ExtraTrees selectors were restricted to 200 CpGs to ease the comparison with *limma*-based feature selection. The maximal number of iterations in L1-based selectors was set to 5000. Other parameters were kept as default.

### Machine learning classifiers

We explored the possibility of applying DNA methylation for depression classification with DL and ML classifiers. The Python module *scikit-learn* [39] was used as a source for ML classifiers. The models included binary logistic regression (no regularization), “ridge” logistic regression (“L2” regularization), “lasso” logistic regression (“L1” regularization), elastic net logistic regression (“L1L2” regularization), decision tree, random forest, support vector machines (SVMs) with linear or radial basis function (RBF) kernels, and AdaBoost. The classifiers were primarily used with default parameters, except for SVMs and several parameters in other models (see Data S1).

### Standalone deep learning classifiers

DL models were selected based on the input tensor properties as well as on the previous architectures that were applied in similar domains of applications. We tried nine different versions of small deep neural network classifiers (Fig S1). In all models, the first layer represents a 1D tensor (for a single participant) with the overall shape (batch, N), where N = 200 is the number of selected CpGs. The last layer in all implementations represents a single node with sigmoid activation. Between the input and output layers, we tried different combinations of hidden layers with regularizations, batch normalization and/or dropout. We also tried different combinations of layer activations. The loss function for all classifiers was represented by a binary cross-entropy.

### Joint autoencoder-classifier models

We investigated the applicability of depression classification with encoded DNA methylation data as was proposed in other areas [32, 33, 68]. These approaches imply the encoding of the DNA methylation data into hidden space with autoencoders before the classification. The training of model components is performed jointly. In these models, the latent space is used both for reconstruction and classification. The autoencoder component of models was implemented either as a fully connected autoencoder or a variational autoencoder (VAE). Each autoencoder consists of an encoder part and a decoder part. A structure of fully-connected autoencoders was based on a sequence of fully-connected layers with N, 128,64, nodes for encoder, and 64,128, N nodes for a decoder. The bottleneck layer is represented by a fully-connected layer with 32 nodes. The bottleneck layer in VAE, in turn, was represented by a sampling layer with 32 nodes. Reconstruction loss functions of all autoencoder types were dependent on the input, and mean squared error loss was used for M-values, whereas binary cross-entropy was used for beta-values.

**Fig. 2:**
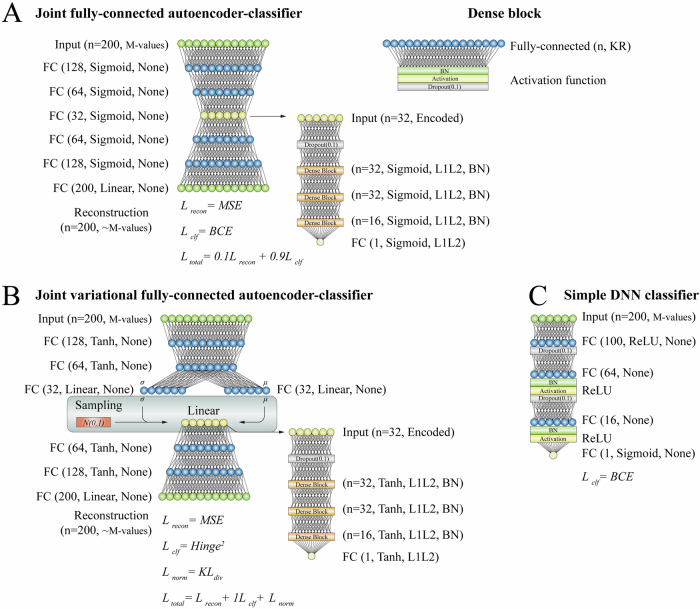
Configurations of deep learning models. This figure shows configuration of deep learning models used to distinguish between depression cases and controls. **A** The resulting configuration of a joint fully-connected autoencoder-classifier (JointAE classifier) and a dense block module. **B** The resulting configuration of a joint variational fully-connected autoencoder-classifier (JointVAE classifier). **C** The resulting configuration of the standalone deep learning classifier (simple DNN classifier). All models were implemented with tensorflow2. Model parameters were obtained after grid search on harmonized data with unbiased feature selection and are specified in the figure. Training for JointAE classifier, JointVAE classifier, and simple DNN classifier was performed for 2000, 2250, and 1000 epochs, respectively. Batch size was set to 128. Learning rate was set to 0.0001. FC fully connected, KR kernel regularization, BN batch normalization, MSE mean squared error, BCE binary cross-entropy.

The classifier part of all models represented a small fully connected neural network with dropout, regularization, batch normalization, and activations treated as *hyperparameters*. The last node of the classifier had either sigmoid or hyperbolic tangent activation for binary cross-entropy and squared hinge losses, respectively. The structure of all final configurations of DL models is shown in Fig. 2. The classification loss (L_clf_) was primarily represented by a binary cross-entropy. The total loss for fully connected autoencoder-classifiers was set as a weighted average of the reconstruction and classification loss functions. The total loss for VAE classifier was formulated as a sum of reconstruction loss, Kullback–Leibler divergence loss, and classification loss scaled by a scalar (*hyperparameter*). Detailed description of DL model preparation could be found in Data S1.

### Model training, optimization, and evaluation

Model training and hyperparameter searches were either performed at the local computer with NVIDIA RTX A5000 GPU or at the dedicated server nodes (with two NVIDIA A100 GPUs) provided by the National Academic Infrastructure for Supercomputing in Sweden (NAISS) at Uppsala Multidisciplinary Center for Advanced Computational Science (UPPMAX).

Model training, optimizations, and preliminary performance evaluations were conducted on a cross-validation (CV) dataset. This dataset included ~84% of the entire data and was composed of five out of eight cohort batches (GSE125105_MPIP, GSE198904_DHRC, GSE72680_GRADY, PSY_SCR, GSE113725_RDE). The independent test set was not used for model optimizations and included all data from the remaining three cohorts (PSY recall, GSE74414_MPIP2, and GSE198904_OBS). PSY recall and GSE74414_MPIP2 were included in the test as these cohort batches represent a population analogue of the respective larger cohorts used in training (PSY_SCR and GSE125105_MPIP). The cohort GSE198904_OBS was allocated to the test set so its total number is ~15% (standard for independent test samples). Data allocation for the independent test sets was strictly based on cohort batches so cohort-level preprocessing and technical batch (based on physically discrete Illumina BeadChips [69]) is not leaked between test set and CV-set. The full distribution of depression cases and controls across cohort batches is available in Data S2.

The optimizer *Adam* [70] was used in the training of DL models. The hyperparameter searches for DNN models were performed with a single threefold cross-validation on the CV dataset using averaged hold-out subset AUCs. Hyperparameter searches for SVMs with a single threefold cross-validation on the CV dataset using averaged hold-out subset AUCs and accuracy in training and test. The evaluation of best-performing classification models was performed via 10 repetitions of threefold cross-validation on the same CV dataset using averaged statistics for hold-out CV subsets. The last model evaluation has been performed on a separate hold-out test set. Effects from different feature selection strategies on model performances were evaluated via 10 repetitions of threefold cross-validation (CV dataset) and in an independent test set using already established model configurations. All DL models were implemented in tensorflow2, ML models were implemented in *scikit-learn*, and analyses were conducted with bash, python, and R (*limma*-based feature selection). The source code for all stages of the work is publicly available at https://github.com/AleksandrVSokolov/depression_ ML_DL.

## Results

### Generated cohort

**Fig. 3:**
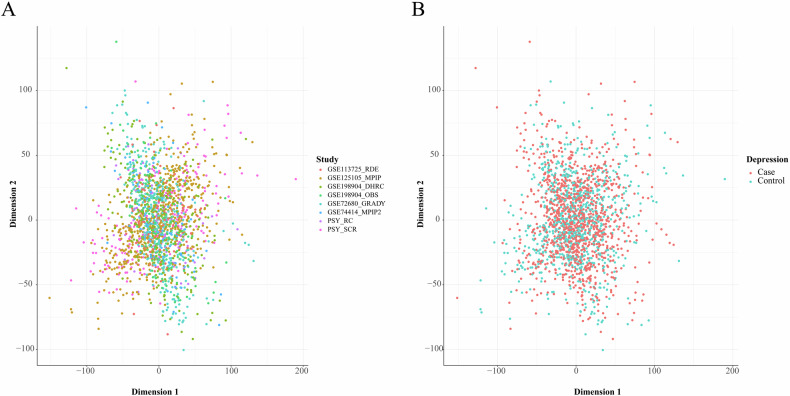
PCA of the pooled data after harmonization. This figure shows the visualization of PCA across the first two principal components obtained in the harmonized dataset. In the sub-plot (**A**), participants (dots) are labeled according to the cohort (batch) of origin, whereas in the sub-plot (**B**), participants are labeled with regards to depression status.

We obtained a combined depression DNA methylation dataset using eight publicly-available cohorts deposited at GEO. All of the included cohorts demonstrated class imbalances, and in some instances, the number of cases/controls was 5–10 fold higher than the opposite class. In the pooled sample, the number of females was almost double than the number of males in both depressed participants and controls. The combined data was skewed toward depressed participants and included 1127 cases and 815 controls (Data S2). However, at the level of the individual cohort, this trend was not consistent and even opposite in some cases, such as in PSY and MPIP2 cohorts. The obtained methylation in the studies was inconsistent depending on the methylation batch/cohort, showing different means and variances (Fig S2A). However, after harmonization, the methylation was stabilized and demonstrated homogeneous distributions (Fig S2B). The PCA visualization of the first two components derived from the harmonized data showed a homogeneous distribution of participants with regards to cohort of origin as well as depression status (Fig. 3A, B).

### Pooled differential methylation analysis

After obtaining the combined harmonized dataset, we performed pooled differential methylation analysis to identify multi-cohort depression-related CpGs. This analysis yielded 20667 CpGs that demonstrated nominally significant associations with depression (Figs. S3–4, Data S3). None of the CpGs passed the FDR correction. Top four hits were close to FDR significance: cg02355787, cg07300292, cg18505978, cg24965479. Interestingly, among 20667 nominally significant CpGs, only 723 had consistent directions for median differences between cases and controls in all cohorts. The Top 200 of these CpGs were used to generate the pre-selected list of predictive features (Data S4) with positive performance bias to obtain *maximal theoretical performance* in the classification models.

**Fig. 4:**
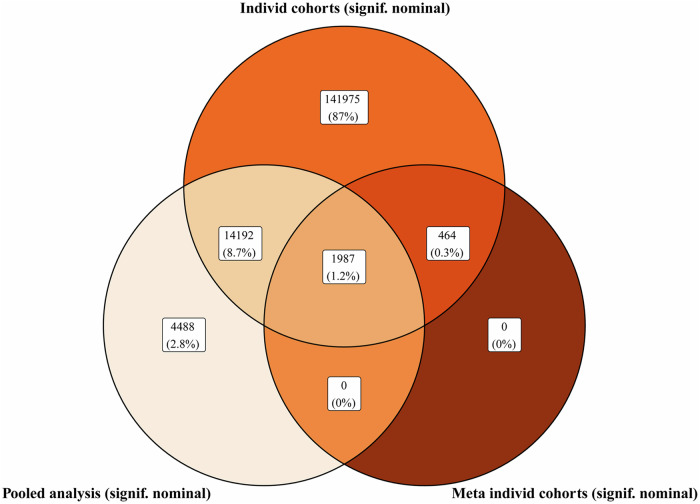
CpGs and the associated analysis. This Venn diagram indicates the number of significant CpGs and their proportions with respect to the type of differential methylation analysis.

### Meta-analysis of cohort-level differential methylation

Being aware that merging individual cohorts and harmonizing data may potentially generate spurious associations and/or erase biological differences among cases and controls, we performed a meta-analysis of differential methylation obtained on individual cohorts before harmonization (batch-level processed data) to compare the results with the pooled analysis. The initial differential methylation on 8 individual cohorts produced 210,760 nominally significant associations, which contained 158,618 unique CpGs (Data S5). Among these, 4422 (4418 unique CpGs) associations passed the FDR correction in the individual cohorts. There was no single CpG site that was nominally significant in all included cohorts, as well as no CpG site that was FDR-significant in more than two cohorts (Data S6). Only one CpG cg25013095 was nominally significant in 6 cohorts, showing matching directions in five. Only 66 CpGs were nominally significant in 4 cohorts with a matching direction of association. Interestingly, only four CpGs were significant at FDR in two cohorts (cg07258897, cg20469261, cg10558233, cg00566320), of which half had matching association directions.

The results from individual cohorts were subsequently meta-analyzed and all CpGs that had at least one nominally significant association were included (158,618 CpGs). The estimated heterogeneity and consistency across cohorts varied greatly with estimated ${I}^{2}\ni \left[0.46 \% ,85.7 \% \right]$ depending on the CpG site tested. In total, 2451 CpGs were found nominally significant in the meta-analysis and none passed the meta-FDR correction (Data S6). Among these, only 29 were FDR-significant in the individual cohorts. We performed a chi-squared test to see if the frequency of FDR-significant CpGs is statistically increased in the list of meta-significant CpGs and found no significant associations between these (*p* = 0.27). Differential methylation for 1554 meta-significant CpGs was nominally significant only in one cohort, whereas 716, 165, 16 were significant in two, three, and four cohorts, respectively. We juxtaposed results from the pooled analysis and meta-analysis to identify CpGs that are associated with depression regardless of the analysis method and found an overlap with 1987 CpGs from both methods (Fig. 4). It should be mentioned that identified differentially-methylated CpGs might have been influenced by confounding arising from smoking. We estimated smoking scores using methylation values before harmonization to see the proportion of the samples having high smoking scores. In the present cohort, only 44 participants (~2%) could be potentially identified as smokers using a strict threshold from Elliott et al. [64] (Data S5). Identified 1987 CpGs were not associated with a smoke-related Aryl Hydrocarbon Receptor Repressor (*AHRR*) [64] gene based on Illumina annotation.

We performed a GO enrichment analysis of genes related to 1987 CpGs, and it yielded no significant biological processes at FDR < 5% with the top nominally enriched biological processes including axon guidance, DNA damage response, membrane processes, and immune-related terms (Data S7-1). We performed an alternative enrichment analysis mapping CpGs to genes as eQTMs with data from the BIOS QTL browser (Data S7-2,3). This enrichment was also only nominally significant to biological processes, but highlighted the terms related to mammary gland morphogenesis and immune system (Data S7-4). Top associated genes that expression was regulated by CpGs in the overlap included *HOTAIRM1, NLGN2, ACSF3, HOXA1, KLHDC7B*, each of those were regulated by at least three eQTMs (Data S7-3).

As methylation affects gene expression through gene regulation, we explored how identified methylation markers are related to chromatin regulatory elements based on the Roadmap Epigenomics project [66]. For this analysis, we identified overrepresented regions both in primary mononuclear cells from blood and prefrontal cortex (Data S7-5). The enrichment analysis identified that CpGs related to increased methylation in depression are located close to gene sequences (near transcription start sites (TSS)) both in blood and prefrontal cortex. Specifically, in blood, both active (associated with gene transcription: Flanking Active TSS, Active TSS) and inactive regions (Flanking Bivalent TSS/Enh, Bivalent/Poised TSS, and Repressed PolyComb) were enriched compared to the reference set of CpGs. A similar pattern was observed in the prefrontal cortex for CpGs with increased methylation. Interestingly, the location of CpGs associated with decreased methylation in depression was enriched with exclusively inactive chromatin states in blood (Quiescent/Low, Weak Repressed PolyComb) and prefrontal cortex (Quiescent/Low, Weak transcription).

### Blood DNA methylation as a predictor for depression

We next utilized the combined depression dataset for evaluation of different classification models. The first step was to generate a cross-validation (CV) dataset that would be used for model optimizations and to obtain averaged evaluations within cohorts since parts of the same cohorts are used for both training and hold-out sets. This dataset included all samples from cohorts GSE125105_MPIP, GSE198904_DHRC, GSE72680_GRADY, PSY_SCR, GSE113725_RDE and contains 84% of the initial data (1626 samples). This dataset, however, could provide slightly positive performance bias since methylation data is initially normalized at a methylation batch level which leads to potential information leakage from hold-out sets to training sets. This bias would imply that obtained performance could only be achieved if training and test data comes from the same methylation analysis batch. Additionally, it has been shown that repeated cross-validation procedures may lead to overfitting during the model optimization process [71]. To obtain a less biased performance estimate, we generated a separate hold-out set that included all samples from PSY recall, GSE74414_MPIP2, and GSE198904_OBS. We used both harmonized and non-harmonized data for the evaluation of classifiers. In the evaluation of the models, we opted to estimate and exclude feature selection bias [72], thus we implemented limma-based feature selection (identification of the differentially methylated CpGs) within each fold of cross-validations on the fold-level training data. However, we also evaluated classifier performance with pre-selected features as this approach could yield maximal theoretical performance on the assumption that consistent reproducible CpGs are known.

**Fig. 5:**
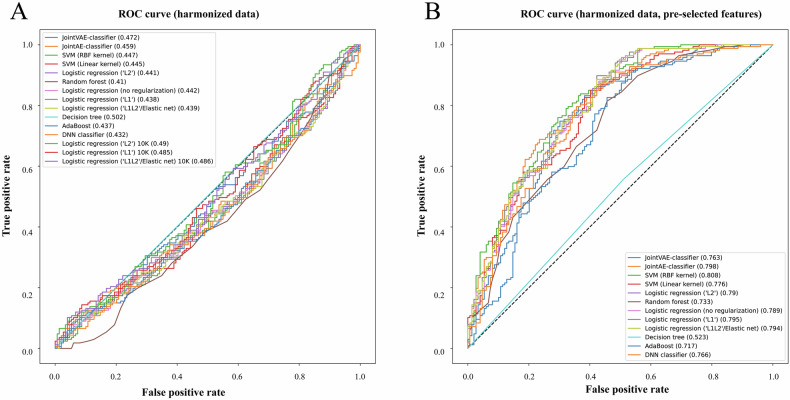
ROC curves for classifiers used on the harmonized data. **A** This sub-figure shows averaged ROCs and associated areas under the curve (AUCs) for classifiers in the testing with the hold-out set with unbiased feature selection. **B** This sub-figure shows averaged ROCs and AUCs for classifiers in the testing with the hold-out set with pre-selected CpGs. All metrics were obtained from data after harmonization.

We first evaluated performance in the harmonized dataset. ML and DL models had distinct complexities and took different times to train. The longest training time was associated with the number of input features, and thus penalized logistic regression models with Top 10,000 CpGs from *limma* took the longest time to train (~15 min per fold). With the 10 repetitions of threefold cross-validation, we estimated the average predictive power of the Top 200 CpGs selected within individual cross-validation folds (unbiased feature selection) (Data S8.1). Models yielded low receiver operating characteristic areas under the curve (AUCs) in the ranges from 0.49 to 0.569 and demonstrated a tendency to overfit training data. The worst performance, in this setting, was demonstrated by a logistic regression without regularization (AUC 0.49) applied on M-values. All penalized regression (“L1”, “L2”, and “Elastic net”) models applied to the top 10,000 depression-related CpGs showed even worse performance (all AUCs < 0.48). In general, all regression-based models demonstrated better performance with Beta-values for methylation. The best performance, in turn, was achieved by random forest models (AUC 0.569). Deep learning models demonstrated average performances with AUCs of 0.529 to 0.538, though joint autoencoder-classifier DL models demonstrated good methylation reconstruction (r > 0.97). As expected, all models performed relatively well with pre-selected features, reaching AUCs up to 0.72 for SVM with RBF kernel applied on M-values (Data S8.1). In the final testing on the hold-out set, however, none of the models showed AUCs > 0.51 when feature selection was within the training process, and only models with external feature selection (positive bias) showed predictive power (AUCs from 0.52 to 0.81) (Data S8.2 and Fig. 5A, B).

**Fig. 6:**
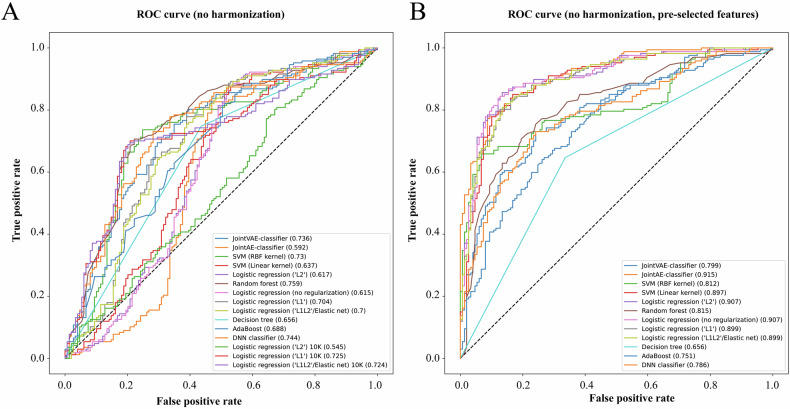
ROC curves for classifiers used on the non-harmonized data. **A** This sub-figure shows ROCs and associated areas under the curve (AUCs) for classifiers in the testing with the hold-out set with unbiased feature selection. **B** This sub-figure shows ROCs and AUCs for classifiers in the testing with the hold-out set with pre-selected CpGs. All metrics were obtained from data before harmonization that was preprocessed at a cohort (batch) level.

Since normalization of training and testing sets together may lead to information leakage as well as data harmonization may remove intrinsic differences in cases and controls across cohorts, we also investigated performances of the same model configurations on the data before harmonization (Data S9). Unexpectedly, we observed dramatically increased performance of classifiers, reaching an AUC of up to 0.73 (random forest applied with M-values) in the CV tests with unbiased feature selection. Other classifiers also showed AUCs in ranges between 0.6 and 0.73. In some of the folds, however, a simple DNN classifier and SVM models showed a propensity to allocate samples from PSY screening and GSE198904_DHRC almost entirely as cases/controls. Interestingly, the performance of models with pre-selected features (positive bias) also improved, reaching AUCs of 0.81 for penalized regression models in CV. Model performances were maintained in the final testing. However, even more model configurations demonstrated increased trends of classifying individual cohorts as one class. Models with unbiased feature selections reached average AUCs of up to 0.76 (random forest with beta values, Fig. 6A). In the biased framework with pre-selected CpGs, in turn, the performance increased even more and some models reached respective AUCs of up to 0.91 (JointAE-classifier, Fig. 6B).

### Feature selection and depression classification

We were primarily evaluating *limma* as a key feature selection strategy in the development of the models as it is one of key approaches for array-based -OMIC analyses [60]. However, alternative feature selections could be used instead of *limma* as it is typically performed in other ML/DL domains. We further explored how performances of established model configurations would change depending on the selected pool of CpGs. We compared the performance of models using Top 5%, Top 1% Top 0.1% of hyper-variable CpGs, as well as using 200 CpGs selected based on L1-penalized linear SVM classifier, L1-penalized logistic regression, ANOVA F-score, and ExtraTrees classifier. We evaluated these strategies, using both CV dataset as well as in the independent test set with and without data harmonization (Data S10 and Fig S6). In the harmonized dataset, feature selection strategies did not have much effect on model performance and random forest with limma-selected 200 CpG showed the highest (albeit low) AUC of 0.569 (10 × 3-fold CV) compared to all other models and feature selection strategies. In the independent testing, however, several models demonstrated increased AUCs (up to 0.61 for random forest with 0.1% hypervariable CpGs), though with still low accuracy (≤54%). In the batch-level data, feature selections demonstrated more noticeable influence on performances in CV. It could be seen that variance-based selection of features (including 5% hyper-variable CpGs and ANOVA F-score) marginally outperformed *limma* in autoencoder-based models and logistic regressions. SVM model with RBF kernel showed significantly worse performance with hypervariable CpGs compared to other strategies (AUC ~ 0.5 with 5% hypervariable CpGs). A selection of CpGs based on ExtraTrees showed AUCs comparable to *limma* in nearly all models. Random forest models performed moderately good (AUC ~ 0.7) regardless of feature selection (Fig S6). In the final testing on non-harmonized data, AdaBoost with ExtraTrees, JointAE-classifier with Top 5% hypervariable CpGs, logistic regression (L1) with ExtraTrees, logistic regression (L2) with ExtraTrees, logistic regression (no reg.) with Top 5% hypervariable CpGs, SVM (linear) with ANOVA F 200, random forest with ExtraTrees, and SVM (RBF) with ExtraTrees outperformed limma-based feature selections (Data S10).

## Discussion

Our analysis across six independent populations in eight batches revealed nominally significant 1987 CpG sites consistently associated with depression in both the mega- and meta-analysis. These CpGs have been associated with axon guidance and immunity providing specific evidence for the potential links between DNA methylation and depression. However, none of the individual CpGs reached nominal significance across all batches, indicating a high inconsistency in DNA methylation patterns related to depression. Our evaluation of 12 machine learning and deep learning strategies together with several feature selection approaches highlights the challenges in classification of depression using DNA methylation data with varied performance dependent on model parameters, feature selection approach, and data preparation. Notably, the data harmonization resulted in low performance (all AUCs < 0.57) without pre-selected features, emphasizing the sensitivity of classifier outcomes to data processing methods. We identified that random forest classifiers outperformed other methods in the harmonized dataset (AUC = 0.569) in the CV, and showed the highest performance using the non-harmonized data with unbiased feature selection (AUC = 0.73 (CV) and 0.79 in final testing) even without optimization. This analysis suggests that random forest models could be the preferred choice for methylation risk scoring due to superior consistent performance and ease of use unless much larger training sets are available. This study underscores the impact of feature selection bias on classification accuracy, cautioning against overreliance on pre-selected features for model construction. Moreover, it emphasizes that the identification of robust CpGs consistently associated with depression could be of higher importance for classifier development than the model architecture itself.

Identified methylation changes within the study show clear association to gene sequences, and CpGs are located in proximity to TSS. Interestingly, increased methylation was located both within active and inactive chromatin regions near TSS, indicating potential suppression of gene expression during cell differentiation and development and also longitudinally. Though exact effects of DNA methylation are still not completely understood, it is generally believed that increased methylation in the promoter regions leads to gene silencing [73]. An interesting observation is that demethylation of CpGs in depression was located in inactive chromatin regions. This may potentially indicate that such changes are non-functional and secondary. Interestingly, these quiescent states were found to be constitutive in most of 127 analyzed epigenomes [66].

At the transcriptional level, identified methylation signatures are related to axon guidance and immunity, which are both fields that are currently pursued in depression [74–77]. Additionally, the involvement of synaptic components and to a lesser degree the immune function was also highlighted by the largest depression GWAS today [4]. The eQTM data indicates that methylation at the identified CpGs is in correlation with expression of the genes that have been already linked to neurological function and disorders, such as *HOTAIRM1* - dopamine neuron differentiation [78], *NLGN2* - depression [79], *ACSF3* [80] and *HOXA1* [81] - autism spectrum disorders, *KLHDC7B* - hearing loss [82] and depression [22]. We emphasize that subsequent analyses on the larger data corpus with more cohorts, when available, could lead to the identification of even more promising CpGs and proteins that could be used as biomarkers for depression as well as additional features for model construction. Importantly, we suggest that these biomarkers should be primarily identified via reproducibility in several studies, as, at least in this work, we observed no association between FDR-significance in one cohort and subsequent significance in meta-analysis. Interestingly, even reproducibility for well-established associations, such as DNA methylation and smoking, could be compromised for probes with unreliable methylation measurement [83].

DNA methylation in blood could predict depression status but with great variability of performances. Data harmonization resulted in a very low performance power of all classifiers (all AUCs < 0.57) with unbiased feature selection. Different data preparation strategies could lead to unexpectedly high differences in performance. In our study, the classifiers performed better with batch-level processed data, showing a > 14% improvement in AUCs and generally exhibited high predictive power across multiple models compared to the harmonized dataset. However, this unexpectedly high performance could be originating not only from the fact that harmonization may erase biological differences but also from the peculiarities of distributions in test sets. We could generally recommend using random forest classifiers for DNA methylation scoring, as this method showed superior performance in the harmonized dataset and in non-harmonized dataset both in CV and in independent tests. More complex models such as “flat” DL approaches and autoencoders did not provide superior performance in our study. These results are consistent with studies pointing out that tree-based models show better performances when applied on tabular data, especially in small datasets, compared to DL models [84]. Potentially, a transfer-learning strategy leveraging the newest DL architecture types (such as transformer) [85] could improve classification performance if applied for DNA methylation scoring and classification on large data corpus, capturing distant interactions between features through the attention mechanism [86].

The achieved AUCs in the current study are comparable with the previously reported depression methylation scores. Random forest models in CV showed similar AUCs to the one obtained in the study by Barbu et al. [37] if harmonized data is considered. Though, Barbu et al. model was adjusted for the genetic information compared to the present analysis. Thus, it would be interesting to see how scores would be affected if depression status was first corrected for genetic information. In the study by Clark et al. [38], authors achieved an AUC of 0.724 in CV using the elastic net classifier. In our study, we achieved a slightly less AUC (0.706) in CV and nearly identical AUCs in the final test when Top 10,000 CpGs from *limma* were used as input for the logistic regression model with elastic net penalty in non-harmonized data. Interestingly, Clark et al. did not subtract genetically-explained variance from the methylations score similar to the present study. Thus, this could explain similar scores obtained in two studies and their relatively high size to the scores from Barbu et al. Lastly, Wang et al. proposed calculation of methylation score based on statistical difference of DNA methylation between promoter and other body region (SIMPO) algorithm [36]. Their proposed model achieved an AUC of 0.6 in the validation set.

Feature selection strategies and feature selection bias [72] may result in a very high observed accuracy. In this study, nearly all classifiers performed *“well”* (some AUCs > 0.8) with the consistent CpGs selected from the pooled analysis regardless of data preparation. This observed bias could potentially explain some of the high AUCs achieved previously where features were pre-selected before model construction [87]. However, this can also indicate that the feature selection strategy (i.e., prior identification of reproducible CpGs in data from multiple studies) may be more important than the structure of the model itself for depression classification. Additionally, different algorithmic feature selection approaches other than *limma*, such as ExtraTrees selection, might be beneficial for classification of depression. However, the random forest model appears to be the most stable regardless of feature preparation (all AUCs > 0.7 in non-harmonized tests). This further favors this approach as it potentially may require less optimization than other models.

It should be noted that this work has limitations. First, depression data comes from different studies where the evaluation of depression cases was not consistent, thus limiting the comparability of samples. We were not able to adjust models for potential confounding effects, such as antidepressant intake and smoking use, as these were not reported in the included cohorts. Even though estimated effects of CpGs should not be confounded by smoking to a large extent, it is not clear how smoking score thresholds from Elliott et al. [64] are applicable to new samples. Some studies had high imbalances in classes that can hinder model training and evaluations. ML models are sensitive to hyperparameters and resulting performance could be further optimized and changed if non-default configurations are used. On the other hand, analyzing multiple cohorts to identify reproducible hits may lead to the discovery of biologically relevant associations, rather than spurious hits that poorly generalize to new studies. Additionally, this study provides unbiased performance estimates of multiple classification models on relatively large (for this domain) real-world data where classes would be imbalanced and evaluation of depression is inconsistent [88–91].

Taken together, this is the first study that provides a comprehensive investigation of DNA methylation across multiple depression cohorts in the whole blood and its applicability for depression detection using multiple ML/DL models. Further investigation of DNA methylation in multiple cohorts will be valuable to identify reproducible methylation signatures using unbiased approaches.

### Supplementary information

Data S1

Data S2

Data S3_S10

Fig S5

Fig S6

## Data Availability

The source code for all stages of the work is publicly-available at https://github.com/AleksandrVSokolov/depression_ ML_DL. Data for the PSY is available upon request to the corresponding author.
