# Supplementary material for: Decoding depression: a comprehensive multi-cohort exploration of blood DNA methylation using machine learning and deep learning approaches
Source: Transl Psychiatry. 2024 Jul 15;14:287. doi: 10.1038/s41398-024-02992-y (PMC11250806; doi:10.1038/s41398-024-02992-y)
Supplement: Supplementary file 1 — Data S1 [file 41398_2024_2992_MOESM1_ESM.pdf]

# Supplementary Materials (Data S1) for

## Decoding depression: a comprehensive multi-cohort exploration of blood DNA methylation using machine learning and deep learning approaches

**Aleksandr V. Sokolov et al.**

***Corresponding author. Email: Helgi.Schioth@neuro.uu.se or Helgi.Schioth@uu.se**

**This PDF file includes:**

Supplementary text

Figures S1 to S4

Descriptions for Figure S5 and Figure S6

Descriptions for Data S2 to S10

**Other Supplementary Materials for this manuscript (not embedded in this file) include the following:**

Data S2 to S10

Figure S5 (provided as separate PDF)

Figure S6 (provided as separate PDF)

**Supplementary Text**

## 2. Supplementary methods

### 2.2.1 PSY screening and recall

The PSY cohort is composed of adolescents from Uppsala, Sweden that were screened for psychiatric disorders with self-administered questionnaires. The depression status of the participants was characterized utilizing the computer-based Development and Well-Being Assessment (DAWBA) questionnaire [1]. A depression band of the questionnaire was used for psychiatric evaluation. This band represents a numeric score ranging from 0 to 5, where each number corresponds with a probability that an individual has depression. These scores have the following probabilities: 0 (<0.1%), 1 (~0.5%), 2 (~3%), 3 (~15%), 4 (~50%), and 5 (>70%) [1]. In this work, all individuals with scores higher than three were classified as the "depressed" group. Participants for the study were recruited in different waves, including the screening phase, and a recall phase >1 year after when participants were invited either to come for follow-up or join the study from the start. DNA methylation data at screening is available for 221 participants. DNA methylation data for 91 participants was available exclusively at the recall stage, not overlapping with screening. The DNAm data was obtained from the whole blood sample and DNAm was assessed with the Illumina Infinium HumanMethylation450 array at screening and Human MethylationEPIC at recall. The extensive information regarding sample collection, DNA extraction, and handling is available elsewhere [2].

### 2.2.2 MPIP cohort 1 and MPIP cohort 2

The cohort GSE125105_MPIP is an epigenome-wide methylation analysis conducted at the Max Planck Institute of Psychiatry, Munich, Germany. Depression status was assessed in the clinical settings according to the Diagnostic and Statistical Manual of Mental Disorders (DSM) IV criteria. Detailed information about cohort, sample preparation, and procedures related to methylation profiling are available in the initial publications [3–6]. All 699 participants were included in the analysis. The cohort GSE74414_MPIP2 was also recruited at the Max Planck Institute of Psychiatry. The study design included an investigation of DNA methylation changes in the context of glucocorticoid receptor stimulation with dexamethasone in MDD cases and controls [7]. Depression was characterized by the 21-item Hamilton Depression Rating Scale (HAMD-21). Patients having at least a moderate depressive episode (HAMD-21 $\geq$ 14) were classified as depressed. For this work, we used baseline subjects prior to stimulation. According to the information from the study investigator, one participant was overlapping with GSE125105 MPIP, and thus was excluded

from the analysis. The resulting sample contained 32 depression "cases" and 49 controls. DNAm data has been obtained from the whole blood with the Illumina Infinium HumanMethylation450 array in both cohorts.

**2.2.3 GRADY cohort**

The cohort GSE72680_GRADY has been created as a result of the Grady Trauma Project study in Atlanta, U.S. This sample includes 422 participants with the objective to investigate associations between genetic and environmental factors with stress. Participants primarily had African-American ethnic backgrounds and came from urban areas with low socioeconomic status [3, 8]. DNAm data has been obtained from the whole blood with the Illumina Infinium HumanMethylation450 array. The cohort was characterized by multiple psychiatric questionnaires, and depression, in particular, was evaluated with the commonly used Beck Depression Inventory (BDI) [9]. BDI represents a numeric score ranging from 0 to 63, where higher numbers indicate stronger depression symptoms. To stratify participants into *depressed* and *non-depressed* groups, we used a composite procedure. First, the BDI score was taken into consideration. We used a strict BDI threshold of 21 for the classification of depression [10], as scores 17-20 could be considered as borderline [11]. Second, we also considered if the participant was under treatment for depression (as this data was available). Thus, the overall procedure included several steps:

1. Participants with BDI ≥ 21 were considered as depressed.
2. Participants undergoing depression treatment were considered as depressed.
3. Participants with BDI < 21 and missing data on depression treatment were excluded from the analysis.
4. Participants not undergoing depression treatment with missing BDI data were excluded from the analysis.
5. All other participants were classified as non-depressed.

The resulting data included 212 cases and 179 controls.

**2.2.4 Royal Devon and Exeter cohort**

The cohort GSE113725_RDE has been collected by the Royal Devon and Exeter (RDE) Tissue Bank, part of the NIHR Exeter Clinical Research Facility in the UK. The initial study was to investigate associations between the history of depression and inflammation in the landscape of DNA methylation [12]. Depression history was self-reported by positively answering a question: ‘Has a doctor ever diagnosed you with depression requiring regular medical treatment?’**.** For the purpose of this work, we only used samples with a history of

depression (n=49) and controls (n=48). DNAm was assessed with the Illumina Infinium HumanMethylation450 array.

#### 2.2.5 Molecular Biomarkers of Antidepressant Response Cohort

The cohort GSE198904_DHRC was conducted by McGill University and Douglas Mental Health University Institute in Canada [13, 14]. Individuals were classified as depressed if they had a diagnosis of a current major depressive episode as per the SCID-I as well as a HAMD-21 score ≥ 20. The control samples were recruited through advertisement. In the initial dataset, many samples were obtained from the same participants, and thus were excluded prior to the analysis. Two participants were excluded due to missing phenotype information. The curated dataset contained 32 control participants, whereas 186 individuals were categorized as cases. Methylation profiling was performed in the whole blood with Illumina Human MethylationEPIC.

#### 2.2.6 Observational clinical study cohort NCT02489305

The cohort GSE198904_OBS is a part of the observational clinical study OBSERVEMDD0001 (NCT02489305) from Janssen Research & Development with the objective to identify blood parameters to predict the near-term relapse of MDD across several locations in the U.S. [15] "Post-depression" patients were classified by having nonpsychotic, recurrent MDD (DSM-V) within the past 24 months, Montgomery Asberg Depression Rating Scale (MADRS) total score ≤ 14, and having an evidence for recent response (within 3 months) to oral antidepressant treatment. The non-affected controls were classified according to self-reported data. Similar to the previous cohort, many samples were obtained from the same individual, and thus were excluded prior to the analysis. After exclusion, the resulting sample contained 115 cases and 29 controls. Methylation profiling was also performed in the whole blood with Illumina Human MethylationEPIC.

### 2.3 DNA methylation data preprocessing

DNAm data for PSY and GSE125105_MPIP was available in the form of raw IDAT files. DNAm data for other cohorts was available from raw BeadStudio intensities in a CSV format. The R package *minfi* was used for data loading and preprocessing and values from IDAT files or CSV files with signals were loaded in R [16]. Methylation intensities were corrected for background using the *noob* method in PSY and GSE125105_MPIP resulting in MethylSet class object [17]. The noob correction was not performed in other cohorts as imported data already represented MethylSet and background correction with *minfi* was not possible. Subsequently, extracted beta values were quantile normalized and corrected for type I and type II probe bias via Beta Mixture Quantile Dilation [18] from the *watermelon* R

package [19]. The normalized beta values then passed through the series of filtering steps. Namely, signals from sex chromosomes as well as probes having a SNP with minor allele frequency >5% within the entire probe sequence or probes with SNPs at a CpG site or at single based extension were discarded. Additionally, all cross-reactive probes identified by Chen et al. [20] and Benton et al [21] were further removed. We filtered participants that had less than 75% of probes with detection p-values < 0.00005 (none were excluded). CpG sites that had less than 75% of samples with detection p-values < 0.01 were removed. Bead batch effect correction was performed with *ComBat f*rom *sva* R package. We used a *minfi*-based implementation of the Houseman algorithm to estimate heterogeneity of white peripheral blood cells [22–24]. We used a regression-based method to adjust methylation values for cell-based heterogeneity [25]. Briefly, methylation value is regressed against estimated cell proportions using a linear model. The obtained residuals (that represent the variance unexplained by cell proportions) are then added to the mean value for a CpG to obtain cell-type adjusted methylation intensity. As the data for methylation was coming from two different arrays, we only performed the analysis for overlapping CpG sites. Thus, every participant in each cohort was characterized by a 1D methylation tensor consisting of 304765 methylation values.

DNAm intensity could be represented as either beta-values ($\beta \in [0, 1]$) or as M-values ($M \in R$), where R denotes real numbers. The equations below denote the calculation of beta-values and M-values respectively from the raw signal intensifies. The 100 represents Illumina's constant to avoid divisions by 0.

$$Beta_i = \frac{max(y_{i,methyl}, 0)}{max(y_{i,unmethyl}, 0) + max(y_{i,methyl}, 0) + 100} \quad (1)$$

$$M_i = log_2(\frac{max(y_{i,methyl}, 0) + 100}{max(y_{i,unmethyl}, 0) + 100}) \quad (2)$$

Both, β and M-values could be used for statistical analyses, however M-values empirically showed better performance in specific tests [26]. In the present work, the application of either β or M-values was considered as a *hyperparameter*. Prior to the analysis, the methylation values were ordered according to their genomic coordinates (cumulative positions) and only CpGs that passed QC in all data batches were kept (304,765 CpGs). Individual cohort-batches were concatenated together to form a single large batch-level processed dataset. Since methylation values in every cohort were centered around different means and showed inconsistent variance, the pooled dataset was further quantile normalized, and stabilized with ComBat (cohort of origin was used as batch). The resulting data

represents a harmonized dataset. We compared depression classifications with and without data harmonization. The quality of cohort-batch correction was inspected by PCA on hypervariable CpGs with difference in beta values > 0.2 between 97.5 and 2.5 percentiles of corresponding beta values. PCA was performed with the R package *FactoMineR*.

**2.4 Differential methylation analyses**

Differential methylation analyses were performed within the R environment. We used both pooled analysis of DNA methylation (data from all cohorts is merged into one) as well as meta-analysis of individual cohorts. The pooled analysis was performed on the harmonized dataset (described above). Differential methylation was assessed using R package *limma* based on linear models with T statistics being moderated by an empirical Bayes framework that is commonly used for microarray data [27]. As DNA methylation and depression could be affected by many potential confounders, we included the age and sex of the participants as covariates in all models since only these variables were available for all samples. In the linear models, depression status (case) was used as the main predictor, covariates included age (numeric), sex (binary), and study factor (8 levels), whereas methylation at a corresponding CpG was treated as a dependent variable. For nominally significant CpGs, we calculated a directional agreement index that corresponds to a fraction of cohorts, where the difference in median methylation in cases vs median methylation in controls had the same sign.

For the meta-analysis of individual cohorts, we used data before harmonization (normalized only at a batch/cohort level). We have performed limma-based modeling on individual cohorts, where models included the same parameters but without a study factor (8 levels). For every nominally significant CpGs in at least one cohort, we calculated the number of occurrences for being nominally significant, significant at the false discovery rate of 5% (FDR), and the directional agreement index for FDR and nominal significances. We performed a meta-analysis of estimated log2 fold changes (Log2FC) for all nominally significant CpGs. We considered this as a minimal requirement and potential indication that such a CpG would have a non-zero effect in the meta-analysis. The limma-estimated Log2FC and corresponding standard errors from eight cohorts were used to fit linear random-effects models with R package *metafor* (function *rma.uni*) for every nominally-significant CpG. Standard errors (SE) in *limma* for log2 fold changes were estimated according to a package developer as specified in the package support forum: https://support.bioconductor.org/p/70175/. The *rma.uni* used log2 fold changes (vector of 8 values) and corresponding sampling variances ($SE^2$, vector of 8 values) as input per CpG.

The heterogeneity $\tau^2$ was estimated using Sidik-Jonkman estimator [28, 29] since the default typically better performing restricted maximum likelihood (REML) was not converging for some of the probes even after 5000 iterations. Study weights were estimated with the default *inverse-variance* method. Modeling was performed individually as one CpG at a time. P-values estimated in the meta-analysis were adjusted using the false discovery rate (FDR) method.

**2.5 Sensitivity analysis and functional analyses**

As the initial phenotypic data does not have reported smoking status, we performed estimation of the smoking score from methylation intensities (before harmonization) to see which proportion of participants could be smokers in the analyzed dataset. We used the R package *EpiSmokEr* [30] to perform estimation of the smoking score based on the methodology initially proposed by Elliott et al [31]. We used two score thresholds, both 17.55 (for European population) and 11.79 (for Asian population) as reported in the initial paper [31]. The first score threshold should be applied as the present study uses cohorts from populations with primarily European ancestry. However, we also compared the score to the threshold in the Asian population to see what could be the highest expected smoking level (as % of study sample) if the score is strict.

The obtained list of overlapping nominally significant CpGs from pooled analysis and meta-analysis was subject to gene ontology enrichment analysis. We used R package missMethyl to perform enrichment analysis that takes into account a gene-length bias and multi-mapped CpGs [32]. We also performed enrichment of CpGs considering them as expression quantitative trait methylation (eQTMs) at FDR < 5% based on the data from the BIOS QTL browser (https://molgenis26.gcc.rug.nl/downloads/biosqtlbrowser/). The initial annotation was modified so genes associated with CpGs were replaced by genes from BIOS QTL. The resulting enrichment was performed as unbiased since mapping to genes was based on expression association rather than genomic position/array design.

The obtained list of overlapping nominally significant CpGs was also mapped to chromatin regulatory elements identified by Roadmap Epigenomics Consortium [33]. Mapping was performed based on two cell/tissue types and included E062 primary mononuclear cells (blood) and E073 prefrontal cortex. A blood sample was used to represent the same sample source as in the current study. Prefrontal cortex was used as it has been

consistently linked to depression biology [34]. After every CpG was mapped to a corresponding regulatory element (per tissue), we performed an enrichment analysis of mappings obtained from 1987 CpGs in comparison to a mapping from all CpGs included in the study (304,765). The enrichment was performed, using R package *clusterProfiler*, and was done separately for up-regulated and down-regulated probes.

### 2.6 Feature selection for depression classification

Since the number of initial features is magnitudes times the number of the participants, we performed a feature selection procedure before training classification models. First, we generated a list of 200 CpGs that represents the Top 200 CpGs from pooled differential methylation analysis with consistent direction for association in all cohorts. This list has a positive predictive bias and was used to evaluate the "*maximal theoretical performance*" of classifiers. To evaluate performance in an unbiased manner, we implemented the feature selection procedure within the model training process. In this setting, we used *limma* to identify the top 200 differentially methylated CpGs obtained exclusively from the training sets (generated within each iteration of cross-validation or the entire validation dataset in the final testing). Linear models in *limma* corresponded to the models used in the pooled differential analysis. For regularized logistic regression models, we also evaluated performance using the Top 10 000 CpGs from *limma* as such models assign zeros to many coefficients and could be used to select the most relevant features.

We compared *limma* with alternative algorithm-based feature selection strategies implemented within the training of models (unbiased feature selection). These strategies were based on built-in functions of the *scikit-learn* [35] module and included variance threshold methods (Top 5%, 1%, or 0.1% of CpGs are used as features), selection of features based on models with L1 regularization (linear support vector classifier and logistic regression), selection based on ANOVA F-value, and selection of CpGs based on ExtraTrees classifier. Variance threshold based selectors identify 15239, 3048, and 305 CpGs (features), respectively. L1-based, ANOVA-based, and ExtraTrees selectors were restricted to 200 CpGs to ease the comparison with *limma*-based feature selection. The maximal number of iterations in L1-based selectors was set to 5000. Other parameters were kept as default. Included tested strategies are common and listed in the *scikit-learn* documentation https://scikit-learn.org/stable/modules/feature_selection.html. Sequential feature selection and recursive feature elimination were not tested as individual participants are represented by 304,765 CpGs.

### 2.7 Machine learning classifiers

We explored the possibility of applying DNA methylation for depression classification with DL and ML classifiers. The python module scikit-learn [35] was used as a source for ML classifiers. The models included binary logistic regression (no regularization), "ridge" logistic regression ("L2" regularization), "lasso" logistic regression ("L1" regularization), elastic net logistic regression ("L1L2" regularization), decision tree, random forest, support vector machines (SVMs) with linear or radial basis function (RBF) kernels, and AdaBoost. The classifiers were primarily used with default parameters with a few exceptions:

1) In SVMs, the kernel type was explicitly specified (either RBF or linear). As SVMs are very sensitive to hyperparameters, we performed a grid-search of a C parameter in SVMs with linear kernel and a search of C and gamma in SVMs with RBF kernel. The models were optimized for performance on M-values in harmonized cross-validation dataset. The best configuration with a linear kernel included a model with C=1, whereas the best model with RBF had the following specifications: C=1, gamma=0.25.

2) In the logistic regressions, the maximal number of iterations was set to 5000 to enable solver convergence, the solver, in turn, was set to "saga", and the elastic net L1 ratio was set to 0.5.

### 2.8 Standalone deep learning classifiers

DL models were selected based on the input tensor properties as well as on the previous architectures that were applied in similar domains of applications. We tried nine different versions of small deep neural network classifiers Figure S1. In all models, the first layer represents a 1D tensor with shape (batch, N), where N=200 is the number of selected CpGs. The last layer in all implementations represents a single node with sigmoid activation. Between the input and output layers, we tried different combinations of hidden layers with regularizations, batch normalization and/or dropout. We also tried different combinations of layer activations. The loss function for all classifiers was represented by a binary cross-entropy.

### 2.9 Joint autoencoder-classifier models

We investigated the applicability of depression classification with encoded DNA methylation data as was proposed in other areas [36–38]. These approaches imply the encoding of the DNA methylation data into hidden space with autoencoders before the classification. The training of model components is performed jointly. In these models, the latent space is used both for reconstruction and classification. The autoencoder component of

models was implemented either as a fully-connected autoencoder or a variational autoencoder (VAE). Each autoencoder consists of an encoder part and a decoder part. A structure of fully-connected autoencoders was based on a sequence of fully-connected layers with N, 128, 64, nodes for encoder, and 64, 128, N nodes for a decoder. The bottleneck is represented by a fully-connected layer with 32 nodes.

The VAE is the type of autoencoder that models a hidden dimension to conform to a specified distribution (usually Gaussian) [39]. The encoder part of VAE included the input layer, followed by fully-connected layers with 128 and 64 nodes respectively. Since VAE models hidden dimension as a normal distribution, the next two layers are specified to perform reparametrization and model the mean $\mu$ and logarithm of variance $log(\sigma^2)$, which then are used to generate a sample from a normal distribution with a sampling layer defined as

$$\sigma = e^{(0.5 + log(\sigma^2))} \tag{3}$$

$$\varepsilon \sim N(0, 1)$$

$$z = \mu + \sigma\varepsilon$$

where ε is a sample from a normal distribution (noise variable) and $z$ is the generated latent space. The bottleneck is represented by a sampling layer with 32 nodes. The decoder part of VAE matches the one from the fully-connected autoencoder.

Reconstruction loss functions of all autoencoder types were dependent on the input, and mean squared error loss was used for M-values, whereas binary cross-entropy was used for beta-values as defined below

$$L_{recon\,(M-val)} = \frac{1}{N}\sum_{i=1}^{N}(j_i - \hat{j}_i)^2 \tag{4}$$

$$L_{recon\,(beta-val)} = -\frac{1}{N}\sum_{i=1}^{N}(j_i log(\hat{j}_i) + (1 - j_i)log(1 - \hat{j}_i)) \tag{5}$$

In both equations, N corresponds to batch size, and $j_i$ denotes a real methylation intensity, whereas $\hat{j}_i$ denotes the reconstructed methylation intensity.

The classifier part of all models represented a small fully-connected neural network with dropout, regularization, batch normalization, and activations treated as *hyperparameters.* The last node of the classifier had either sigmoid or hyperbolic tangent activation for binary cross-entropy and squared hinge losses, respectively. The structure of all final configurations

of DL models is shown in Figure 2. The classification loss ($L_{clf}$) was primarily represented by a binary cross-entropy:

$$L_{clf} = -\frac{1}{N}\sum_{i=1}^{N}(y_i log(p_i) + (1 - y_i)log(1 - p_i)) \tag{6}$$

where N corresponds to batch size, $y_i$ denotes a real class, whereas $p_i$ denotes a predicted class. In some architectures, we additionally tested a squared hinge loss as a classification loss. The total loss for fully-connected autoencoder-classifier was set as a weighted average of the two loss-functions:

$$L_{total} = aL_{recon} + (1 - a)L_{clf} \tag{7}$$

where $a \in [0, 1]$ and treated as a *hyperparameter.*

The VAE, however, requires to have an additional loss function to ensure that hidden dimension $z$ conforms to a normal distribution. This loss is derived from the Kullback–Leibler divergence and formulated by the following equation:

$$KL_{loss} = -D_{KL} = -\frac{1}{2}\sum_{i=1}^{N}(1 + log(\sigma_i^2) - \mu_i^2 - \sigma_i^2) \tag{8}$$

where N represents the batch size, $log(\sigma_i^2)$ and $\mu_i$ represent parameters for the sampling layer as discussed above. The total loss for VAE classifier, in turn was formulated as a sum of reconstruction loss, $KL_{loss}$, and classification loss scaled by a parameter $a$.

$$L_{total, VAE} = L_{recon} + KL_{loss} + aL_{clf} \tag{9}$$

$a \in [0, + inf)$ and was treated as a *hyperparameter.*

**2.10 Model training and evaluation**

Model training and hyperparameter searches were either performed at the local computer with NVIDIA RTX A5000 GPU or at the dedicated server nodes (with two NVIDIA A100 GPUs) provided by the National Academic Infrastructure for Supercomputing in Sweden (NAISS) at Uppsala Multidisciplinary Center for Advanced Computational Science (UPPMAX).

Model training and optimizations and preliminary performance evaluations were conducted on a cross-validation (CV) dataset. This dataset included ~84% of the entire data and was composed of five out of eight cohort-batches (GSE125105_MPIP, GSE198904_DHRC, GSE72680_GRADY, PSY_SCR, GSE113725_RDE). The independent test set was not used for model optimizations and included all data from the remaining three cohorts (PSY recall, GSE74414_MPIP2, and GSE198904_OBS). PSY recall and

GSE74414_MPIP2 were included in the test as these cohort-batches represent a population analogue of the respective larger cohorts used in training (PSY_SCR and GSE125105_MPIP). The cohort GSE198904_OBS was allocated to the test set so its total number is ~ 15% (standard for independent test samples). Data allocation for the independent test sets was strictly based on cohort-batches so cohort-level preprocessing and technical batch (based on physically discrete Illumina BeadChips [40]) is not leaked between test set and CV-set.

The optimizer *Adam* [41] was used in the training of DL models. The hyperparameter searches were performed with a single 3-fold cross-validation on the CV dataset using averaged hold-out subset AUCs. The evaluation of best-performing classification models was performed via 10 repetitions of 3-fold cross-validation on the same CV dataset using averaged statistics for hold-out CV subsets. The last model evaluation has been performed on a separate hold-out test set. All DL models were implemented in tensorflow2, ML models were implemented in scikit-learn, and analyses were conducted with bash, python, and R (*limma*-based feature selection). The source code for all stages of the work is publicly-available at https://github.com/AleksandrVSokolov/depression_ML_DL

## References


1. Goodman A, Heiervang E, Collishaw S, Goodman R (2011) The “DAWBA bands” as an ordered-categorical measure of child mental health: description and validation in British and Norwegian samples. Soc Psychiatry Psychiatr Epidemiol 46:521–532
2. Sokolov AV, Manu D-M, Nordberg DOT, Boström ADE, Jokinen J, Schiöth HB (2023) Methylation in MAD1L1 is associated with the severity of suicide attempt and phenotypes of depression. Clin Epigenet 15:1
3. Zannas AS, Jia M, Hafner K, et al (2019) Epigenetic upregulation of FKBP5 by aging and stress contributes to NF-κB-driven inflammation and cardiovascular risk. Proc Natl Acad Sci U S A 116:11370–11379
4. Heck A, Lieb R, Ellgas A, et al (2009) Investigation of 17 candidate genes for personality traits confirms effects of the HTR2A gene on novelty seeking. Genes Brain Behav 8:464–472
5. Kohli MA, Lucae S, Saemann PG, et al (2011) The neuronal transporter gene SLC6A15 confers risk to major depression. Neuron 70:252–265
6. Lucae S, Salyakina D, Barden N, et al (2006) P2RX7, a gene coding for a purinergic ligand-gated ion channel, is associated with major depressive disorder. Hum Mol Genet 15:2438–2445
7. Zannas AS, Arloth J, Carrillo-Roa T, et al (2015) Lifetime stress accelerates epigenetic aging in an urban, African American cohort: relevance of glucocorticoid signaling. Genome Biol 16:266
8. Binder EB, Bradley RG, Liu W, et al (2008) Association of FKBP5 polymorphisms and childhood abuse with risk of posttraumatic stress disorder symptoms in adults. JAMA 299:1291–1305
9. Beck AT, Ward CH, Mendelson M, Mock J, Erbaugh J (1961) An inventory for measuring depression. Arch Gen Psychiatry 4:561–571
10. Holtzheimer PE, Veitengruber J, Wang CC, Krows M, Thiede H, Wald A, Roy-Byrne P (2010) Utility of the Beck Depression Inventory to screen for and track depression in injection drug users seeking hepatitis C treatment. Gen Hosp Psychiatry 32:426–432

11. Kerr LK, Kerr LD (2001) Screening tools for depression in primary care: the effects of culture, gender, and somatic symptoms on the detection of depression. West J Med 175:349–352
12. Crawford B, Craig Z, Mansell G, et al (2018) DNA methylation and inflammation marker profiles associated with a history of depression. Hum Mol Genet 27:2840–2850
13. Sun Y, Drevets W, Turecki G, Li QS (2020) The relationship between plasma serotonin and kynurenine pathway metabolite levels and the treatment response to escitalopram and desvenlafaxine. Brain Behav Immun 87:404–412
14. Ju C, Fiori LM, Belzeaux R, et al (2019) Integrated genome-wide methylation and expression analyses reveal functional predictors of response to antidepressants. Transl Psychiatry 9:254
15. Li QS, Morrison RL, Turecki G, Drevets WC (2022) Meta-analysis of epigenome-wide association studies of major depressive disorder. Sci Rep 12:18361
16. Aryee MJ, Jaffe AE, Corrada-Bravo H, Ladd-Acosta C, Feinberg AP, Hansen KD, Irizarry RA (2014) Minfi: a flexible and comprehensive Bioconductor package for the analysis of Infinium DNA methylation microarrays. Bioinformatics 30:1363–1369
17. Triche TJ, Weisenberger DJ, Van Den Berg D, Laird PW, Siegmund KD (2013) Low-level processing of Illumina Infinium DNA Methylation BeadArrays. Nucleic Acids Res 41:e90
18. Teschendorff AE, Marabita F, Lechner M, Bartlett T, Tegner J, Gomez-Cabrero D, Beck S (2013) A beta-mixture quantile normalization method for correcting probe design bias in Illumina Infinium 450 k DNA methylation data. Bioinformatics 29:189–196
19. Pidsley R, Y Wong CC, Volta M, Lunnon K, Mill J, Schalkwyk LC (2013) A data-driven approach to preprocessing Illumina 450K methylation array data. BMC Genomics 14:293
20. Chen Y, Lemire M, Choufani S, Butcher DT, Grafodatskaya D, Zanke BW, Gallinger S, Hudson TJ, Weksberg R (2013) Discovery of cross-reactive probes and polymorphic CpGs in the Illumina Infinium HumanMethylation450 microarray. Epigenetics 8:203–209
21. Benton MC, Johnstone A, Eccles D, Harmon B, Hayes MT, Lea RA, Griffiths L, Hoffman EP, Stubbs RS, Macartney-Coxson D (2015) An analysis of DNA methylation in human adipose tissue reveals differential modification of obesity genes before and after gastric bypass and weight loss. Genome Biol 16:8
22. Yousefi P, Huen K, Quach H, Motwani G, Hubbard A, Eskenazi B, Holland N (2015) Estimation of blood cellular heterogeneity in newborns and children for epigenome-wide association studies. Environ Mol Mutagen 56:751–758
23. Houseman EA, Accomando WP, Koestler DC, Christensen BC, Marsit CJ, Nelson HH, Wiencke JK, Kelsey KT (2012) DNA methylation arrays as surrogate measures of cell mixture distribution. BMC Bioinformatics 13:86
24. Jaffe AE, Irizarry RA (2014) Accounting for cellular heterogeneity is critical in epigenome-wide association studies. Genome Biol 15:R31
25. Jones MJ, Islam SA, Edgar RD, Kobor MS (2017) Adjusting for Cell Type Composition in DNA Methylation Data Using a Regression-Based Approach. Methods Mol Biol 1589:99–106
26. Du P, Zhang X, Huang C-C, Jafari N, Kibbe WA, Hou L, Lin SM (2010) Comparison of Beta-value and M-value methods for quantifying methylation levels by microarray analysis. BMC Bioinformatics 11:587
27. Ritchie ME, Phipson B, Wu D, Hu Y, Law CW, Shi W, Smyth GK (2015) limma powers differential expression analyses for RNA-sequencing and microarray studies. Nucleic Acids Res 43:e47
28. Langan D, Higgins JPT, Jackson D, Bowden J, Veroniki AA, Kontopantelis E, Viechtbauer W, Simmonds M (2019) A comparison of heterogeneity variance estimators in simulated random-effects meta-analyses. Res Synth Methods 10:83–98
29. Sidik K, Jonkman JN (2005) Simple Heterogeneity Variance Estimation for Meta-Analysis. Journal of the Royal Statistical Society Series C: Applied Statistics 54:367–384
30. Bollepalli S, Korhonen T, Kaprio J, Anders S, Ollikainen M (2019) EpiSmokEr: a robust classifier to determine smoking status from DNA methylation data. Epigenomics 11:1469–1486
31. Elliott HR, Tillin T, McArdle WL, Ho K, Duggirala A, Frayling TM, Davey Smith G, Hughes AD, Chaturvedi N, Relton CL (2014) Differences in smoking associated DNA methylation patterns in South Asians and Europeans. Clin Epigenetics 6:4
32. Maksimovic J, Oshlack A, Phipson B (2021) Gene set enrichment analysis for genome-wide

DNA methylation data. Genome Biol 22:173
33. Roadmap Epigenomics Consortium, Kundaje A, Meuleman W, et al (2015) Integrative analysis of 111 reference human epigenomes. Nature 518:317–330
34. Hare BD, Duman RS (2020) Prefrontal cortex circuits in depression and anxiety: contribution of discrete neuronal populations and target regions. Mol Psychiatry 25:2742–2758
35. Pedregosa F, Varoquaux G, Gramfort A, et al (2012) Scikit-learn: Machine Learning in Python. https://doi.org/10.48550/ARXIV.1201.0490
36. Macías-García L, Martínez-Ballesteros M, Luna-Romera JM, García-Heredia JM, García-Gutiérrez J, Riquelme-Santos JC (2020) Autoencoded DNA methylation data to predict breast cancer recurrence: Machine learning models and gene-weight significance. Artif Intell Med 110:101976
37. Chen L, Saykin AJ, Yao B, Zhao F, Alzheimer's Disease Neuroimaging Initiative (ADNI) (2022) Multi-task deep autoencoder to predict Alzheimer's disease progression using temporal DNA methylation data in peripheral blood. Comput Struct Biotechnol J 20:5761–5774
38. Levy JJ, Titus AJ, Petersen CL, Chen Y, Salas LA, Christensen BC (2020) MethylNet: an automated and modular deep learning approach for DNA methylation analysis. BMC Bioinformatics 21:108
39. Kingma DP, Welling M (2013) Auto-Encoding Variational Bayes. https://doi.org/10.48550/ARXIV.1312.6114
40. Ross JP, van Dijk S, Phang M, Skilton MR, Molloy PL, Oytam Y (2022) Batch-effect detection, correction and characterisation in Illumina HumanMethylation450 and MethylationEPIC BeadChip array data. Clin Epigenetics 14:58
41. Kingma DP, Ba J (2014) Adam: A Method for Stochastic Optimization. https://doi.org/10.48550/ARXIV.1412.6980

## Supplementary Figures S1 to S4

## Figure S1

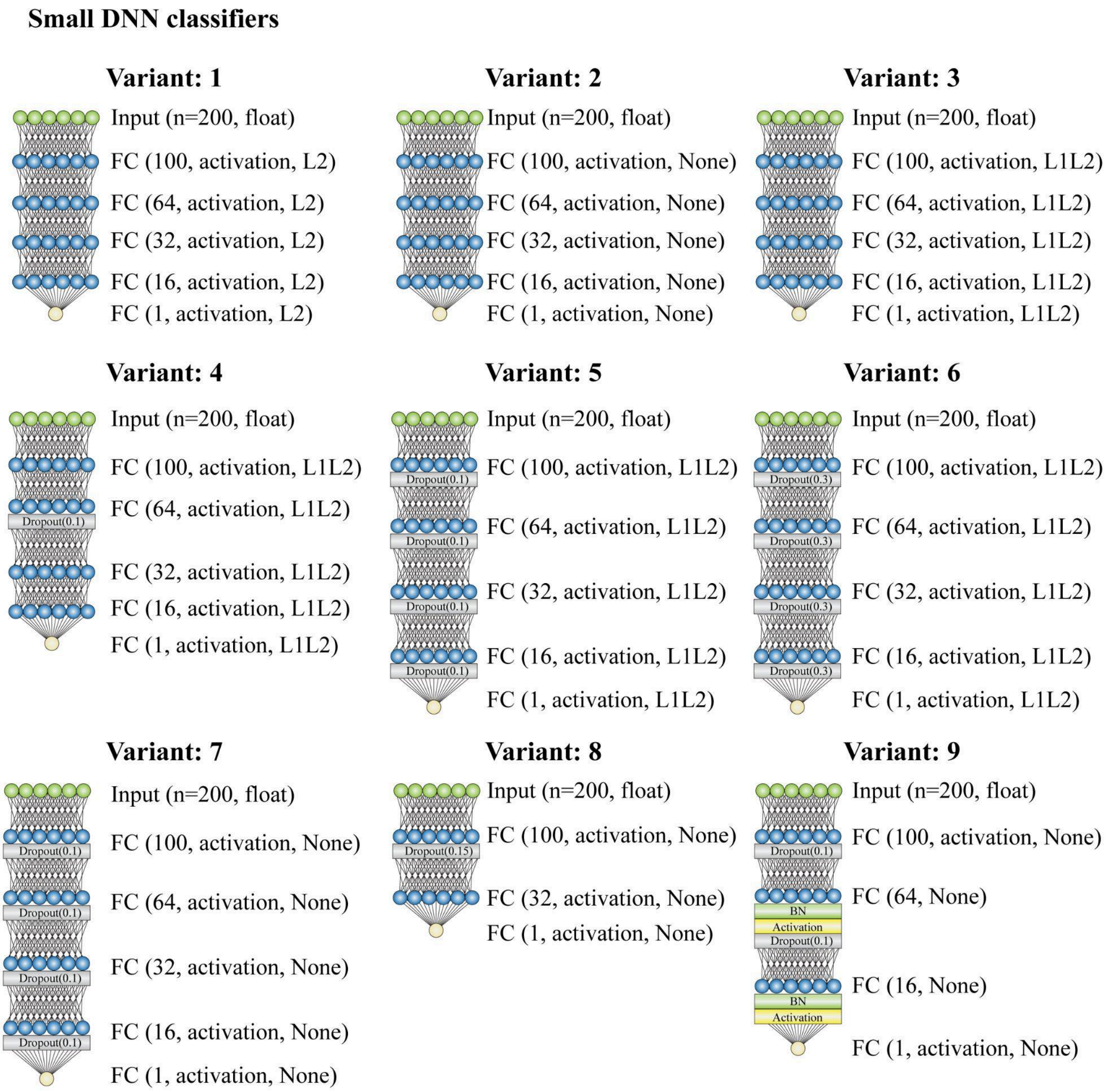


This figure provides schematic representations of nine tested versions of small DNN classifiers in the grid search manner. Each classifier was tested with different combinations of hyperparameters that included activation functions for inner layers of the network (ReLU, sigmoid, or tanh), type of input CpGs (beta-value or M-value), learning rate (0.0001, 0.00001). All networks were implemented with tensorflow. Each model was trained for 2000 epochs during grid search. Classification loss function was set to a binary cross-entropy. Batch size was set to 128.

**Figure S2**

A

B

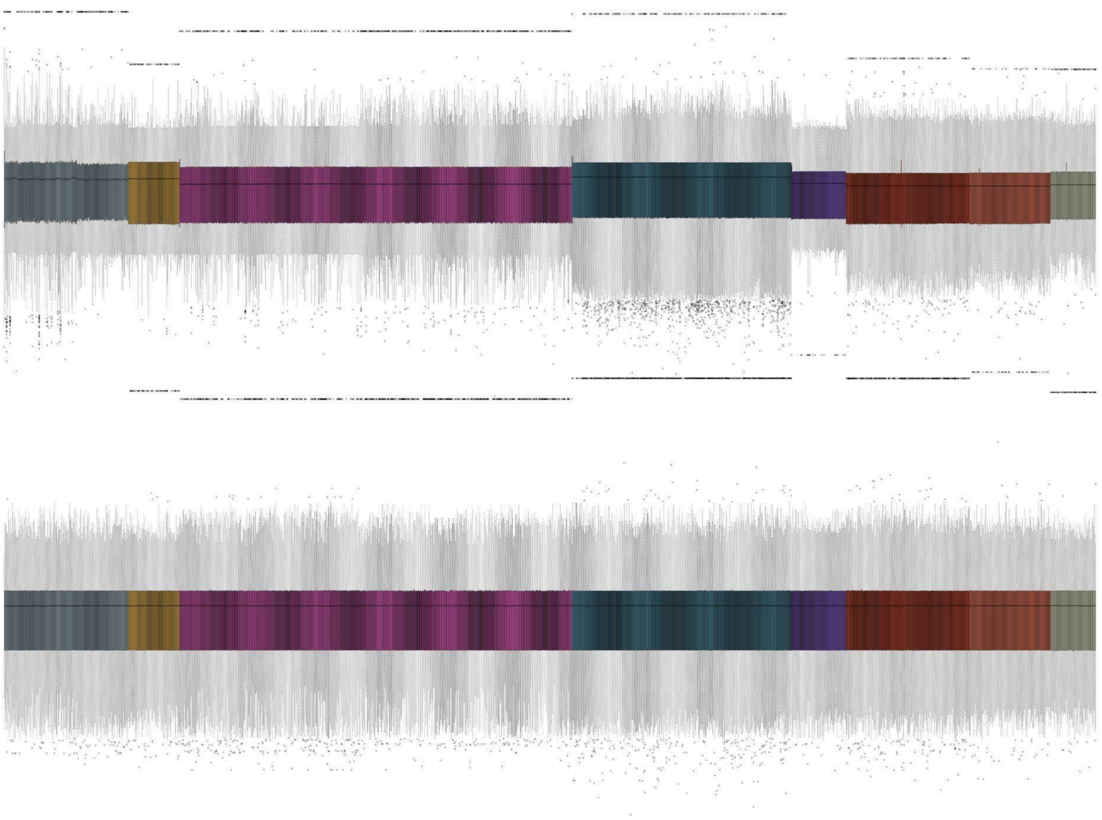

This figure demonstrates distribution of M-values before (data preprocessing at a cohort level) and after cohort harmonization via quantile normalization followed by ComBat.

**Figure S3**

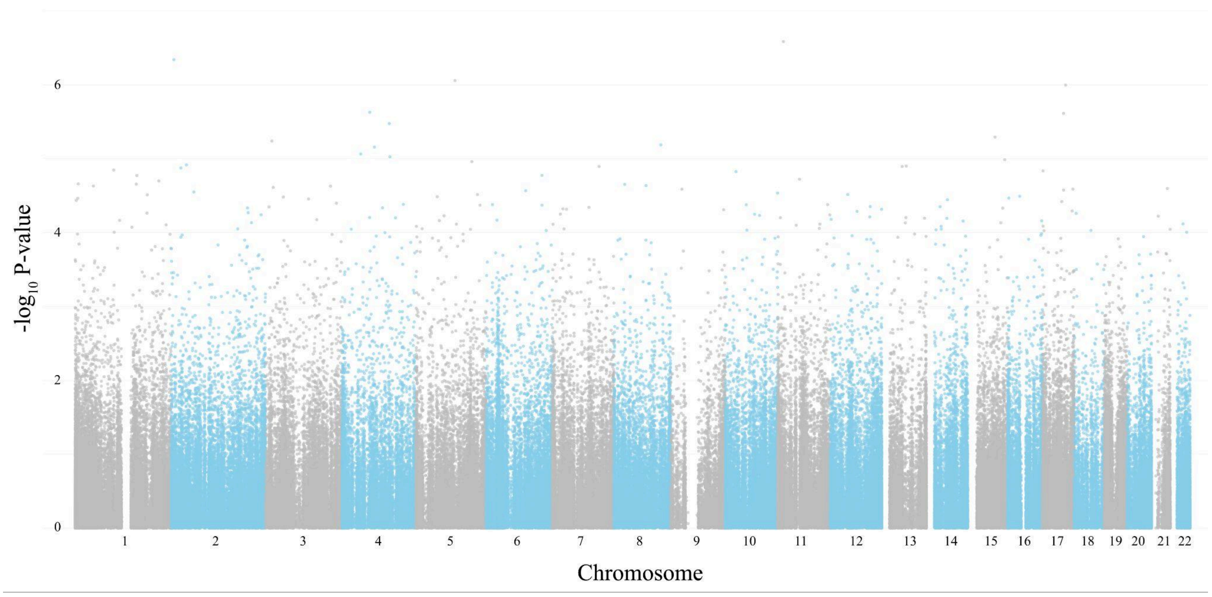


This figure shows the Manhattan plot for pooled differential methylation analysis with limma. None of the probes passed the correction with the false discovery rate < 5%.

**Figure S4**

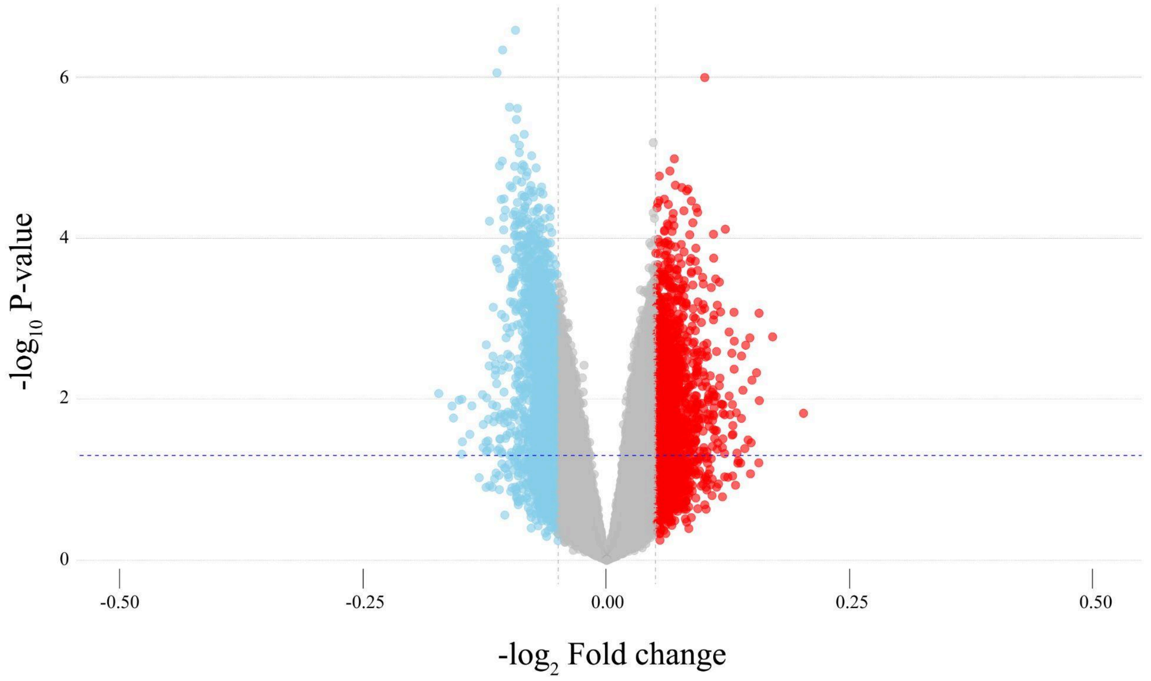


This figure shows the volcano plot for pooled differential methylation analysis with limma. None of the probes passed the correction with the false discovery rate < 5%. In the figures, a threshold of 0.05 for $\log_2$ fold change was set for illustrative purposes. Blue horizontal lines indicate a threshold for nominal significance $\log_2(0.05)$.

**Descriptions for Fig. S5 and Fig. S6**

**Figure S5.** This figure shows estimated smoking scores using R package *EpiSmokEr*. We used data without harmonization (batch-level data). Smoking scores were calculated individually in each cohort-batch, using CpGs that passed quality control steps. Blue line indicates a threshold of 11.79, whereas a red line shows the threshold of 17.55. Both thresholds are from the original publication by Elliott et al.

**Figure S6.** This figure shows the performance of classifiers in relation to feature selection strategies. Y-axis indicates AUC score calculated in the hold-out set for each fold of 3-fold cross-validation. X-axis indicates a feature selection strategy. The first column corresponds to the feature selection by *limma.* Facet axis indicates a classifier model (final configuration). All estimations were performed using 10 repetitions of 3-fold cross validation. Methylation intensity was used as M-values. Each AUC score corresponds to individual fold, and thus every combination of the classifier model and feature selection strategy is represented by 30 AUC values.

**Descriptions for Data S2 to S9**

**Data S2 -** This table displays information on the final dataset, included cohort, and main demographics used in the analysis. Categorical variables are shown as counts and respected percent proportions. Numeric variables are shown as mean ± standard deviation, respected minimum, and maximum.

**Data S3 -** This table displays the results of pooled differential methylation analysis using limma for probes with a nominal p-value < 0.05. Cohort agreement was calculated per CpG by comparing the respective median methylation of "cases" to the respective median methylation of "controls." A positive value indicates that the median in cases was higher than in controls, whereas a negative value indicates the opposite. The prevailing direction is presented in the table. The agreement index was calculated by dividing the number of cohorts with a prevailing direction for association by the total number of cohorts.

**Data S4** - This file shows the list of Top 200 CpGs with consistent directions for associations in all cohorts (median difference) from Data S2 that was used for classifier evaluation with positive performance bias.

**Data S5** - This table shows results for differential methylation analysis with limma performed separately in individual cohorts for probes with a $\text{p-value}_{\text{nominal}} < 0.05$. $Log_2FC$ is shown as a 95% confidence interval. Standard errors for $Log_2FC$ were calculated using limma.

**Data S6** - This table contains significance statistics for individual CpG sites ($\text{p-value}_{\text{nominal}} <$ 0.05 in at least one cohort) as well as an associated meta-analysis. Nominal.pos indicates the number of positive ($Log_2FC$ "Cases" vs "Controls") associations that passed the nominal significance threshold. Nominal.neg indicates the number of negative ($Log_2FC$ "Cases" vs "Controls") associations that passed the nominal significance threshold. Nominal score was calculated as (Nominal.pos + Nominal.neg)/8. Nominal.agreement was calculated as max(Nominal.pos, Nominal.neg)/(Nominal.pos + Nominal.neg). Nominal count equals Nominal.pos + Nominal.neg. Statistics for FDR express the same but for FDR-significant CpGs. meta_LFc indicates meta-calculated $Log_2FC$ for a CpG using linear random effects model. Meta_se indicates the standard error, meta_pval indicates the associated $\text{p-value}_{\text{nominal}}$ for estimated meta_LFcs. Other column show the following: tau2 - τ2 statistic or estimated amount of (residual) heterogeneity, I2 - $I^2$ statistic, H2 - H2 statistic, Q - test statistic of the test for (residual) heterogeneity, Q.p - p-value for Q. meta_FDR indicates meta_pval adjusted with false discovery rate correction. Meta-analysis statistics were calculated using the R package metafor

**Data S7** - **1)** This table shows gene ontology (biological processes) enrichment analysis of 1987 overlapping CpGs taking into the account gene length bias and probes annotated to multiple genes **2)** This table provides a list of CpG-Transcript (eQTM) associations in whole blood at FDR < 5% from BIOS QTL browser. **3)** This table shows affected gene and the number of associated CpG sites from eQTM table **4)** This table shows gene ontology (biological processes) enrichment analysis of 1987 overlapping CpGs where each CpG was mapped to a gene through eQTM association but not through its coordinates. 5) This table shows overrepresentation analysis for chromatin regulatory elements based on the Roadmap Epigenomics Project. State names and description are provided based on the information here: https://egg2.wustl.edu/roadmap/web_portal/chr_state_learning.html#core_15state.

Analysis considered two tissues separately: E073 Pre-frontal cortex, E062 Primary mononuclear cells (blood).

**Data S8** - These tables show results for evaluation of classification models in the cross-validation tests and evaluation in the final hold-out set using data after harmonization

**Data S9** - These tables show results for evaluation of classification models in the cross-validation tests and evaluation in the final hold-out set using data without harmonization

**Data S10** - These tables show results for evaluation of classification models and feature selection strategies in the cross-validation tests and evaluation in the final hold-out set. All evaluations were performed on M-values using both harmonized data and data without harmonization (batch-level data).
