# Supplementary material for: Decoding depression: a comprehensive multi-cohort exploration of blood DNA methylation using machine learning and deep learning approaches
Source: Transl Psychiatry. 2024 Jul 15;14:287. doi: 10.1038/s41398-024-02992-y (PMC11250806; doi:10.1038/s41398-024-02992-y)
Supplement: Supplementary file 2 — Data S2 [file 41398_2024_2992_MOESM2_ESM.pdf]

Data S2

	Controls	Cases
**PSY (screen)**		
**221 participants**		
**Depression status**	Control: 197 (100%)	Case: 24 (100%)
**Sex**	Male: 54 (27.4%)<br>Female: 143 (72.6%)	Male: 2 (8.3%)<br>Female: 22 (91.7%)
**Age**	15.45 ± 0.63<br>Min: 14, Max: 17	15.42 ± 0.65<br>Min: 14, Max: 16
**PSY (recall)**		
**91 participants**		
**Depression status**	Control: 71 (100%)	Case: 20 (100%)
**Sex**	Male: 25 (35.2%)<br>Female: 46 (64.8%)	Male: 2 (10%)<br>Female: 18 (90%)
**Age**	17.51 ± 0.83<br>Min: 15, Max: 20	17.15 ± 0.81<br>Min: 16, Max: 18
**GSE125105 (MPIP1)**		
**699 participants**		
**Depression status**	Control: 210 (100%)	Case: 489 (100%)
**Sex**	Male: 84 (40%)<br>Female: 126 (60%)	Male: 228 (46.6%)<br>Female: 261 (53.4%)
**Age**	49.49 ± 13.32<br>Min: 19, Max: 79	45.64 ± 13.89<br>Min: 17, Max: 87
**GSE72680 (GRADY)**		
**391 participants**		
**Depression status**	Control: 179 (100%)	Case: 212 (100%)
**Sex**	Male: 58 (32.4%)<br>Female: 121 (67.6%)	Male: 55 (25.9%)<br>Female: 157 (74.1%)
**Age**	40.36 ± 13.76<br>Min: 18, Max: 74	42.81 ± 11.21<br>Min: 18, Max: 70
**GSE113725 (RDE)**		
**97 participants**		
**Depression status**	Control: 48 (100%)	Case: 49 (100%)

**Sex**	Male: 12 (25%)<br>Female: 36 (75%)	Male: 13 (26.5%)<br>Female: 36 (73.5%)
**Age**	45.5 ± 9.99<br>Min: 20, Max: 60	45.96 ± 9.36<br>Min: 23, Max: 61
**GSE198904 (DHRC)**		
**218 participants**		
**Depression status**	Control: 32 (100%)	Case: 186 (100%)
**Sex**	Male: 10 (31.2%)<br>Female: 22 (68.8%)	Male: 75 (40.3%)<br>Female: 111 (59.7%)
**Age**	46.62 ± 14.06<br>Min: 21, Max: 68	39.15 ± 12.15<br>Min: 18, Max: 68
**GSE198904 (OBS)**		
**144 participants**		
**Depression status**	Control: 29 (100%)	Case: 115 (100%)
**Sex**	Male: 8 (27.6%)<br>Female: 21 (72.4%)	Male: 23 (20%)<br>Female: 92 (80%)
**Age**	42.9 ± 11.35<br>Min: 23, Max: 63	42.49 ± 11.86<br>Min: 18, Max: 63
**GSE74414 (MPIP2)**		
**81 participants**		
**Depression status**	Control: 49 (100%)	Case: 32 (100%)
**Sex**	Male: 37 (75.5%)<br>Female: 12 (24.5%)	Male: 20 (62.5%)<br>Female: 12 (37.5%)
**Age**	34.43 ± 12.64<br>Min: 21, Max: 59	43.94 ± 14.59<br>Min: 21, Max: 71
**Total**		
**1942 participants**		
**Depression status**	Control: 815 (100%)	Case: 1127 (100%)
**Sex**	Male: 288 (35.3%)<br>Female: 527 (64.7%)	Male: 418 (37.1%)<br>Female: 709 (62.9%)
Age	34.98 ± 17.55<br>Min: 14, Max: 79	42.53 ± 13.75<br>Min: 14, Max: 87
