# Supplementary figures and images for: Decoding depression: a comprehensive multi-cohort exploration of blood DNA methylation using machine learning and deep learning approaches
Source: Transl Psychiatry. 2024 Jul 15;14:287. doi: 10.1038/s41398-024-02992-y (PMC11250806; doi:10.1038/s41398-024-02992-y)

Estimated smoking score vs cohort-batch (cohort-level preprocessing)

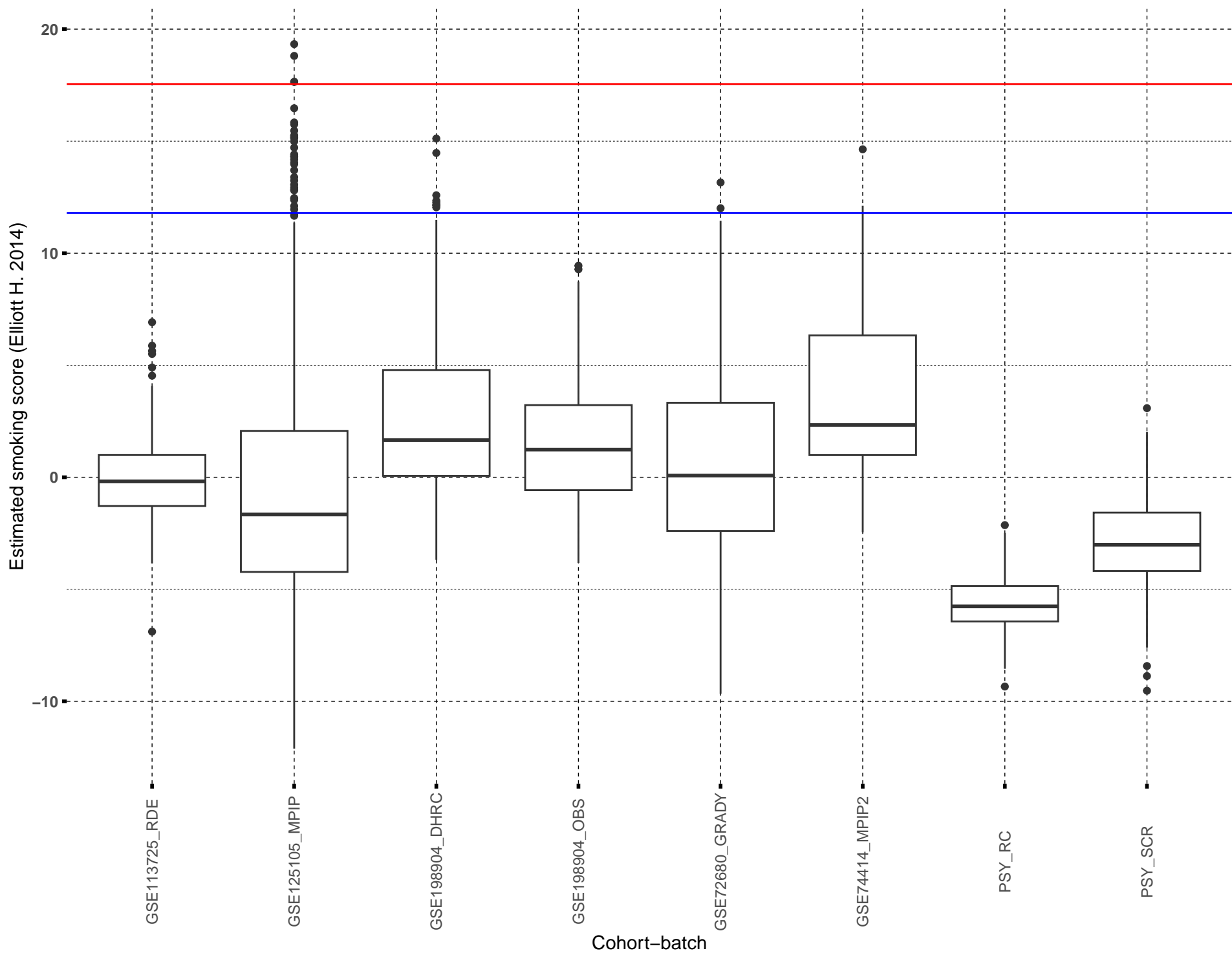

Supplement: Supplementary file 4 — Fig S5 [file 41398_2024_2992_MOESM4_ESM.pdf]

Feature selection strategies in 10x3-fold CV on non-harmonized data

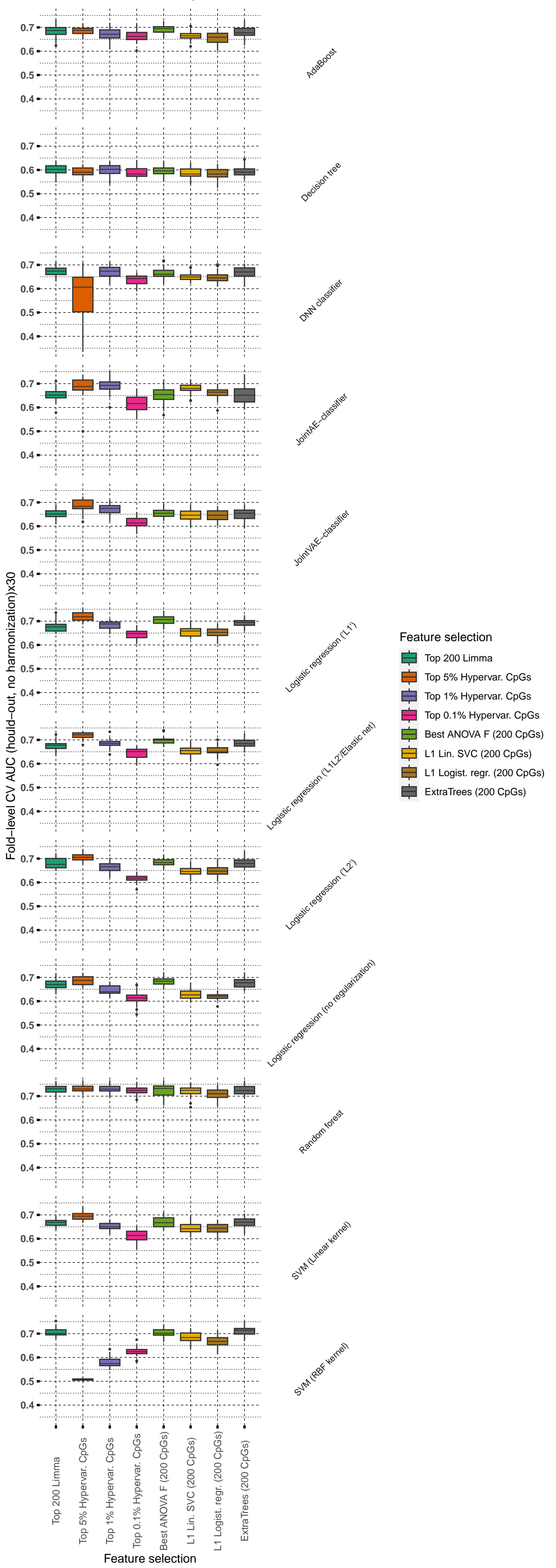

Supplement: Supplementary file 5 — Fig S6 [file 41398_2024_2992_MOESM5_ESM.pdf]
